# Pharmacokinetic and -dynamic modelling of G-CSF derivatives in humans

**DOI:** 10.1186/1742-4682-9-32

**Published:** 2012-07-30

**Authors:** Markus Scholz, Sibylle Schirm, Marcus Wetzler, Christoph Engel, Markus Loeffler

**Affiliations:** 1 Institute for Medical Informatics, Statistics and Epidemiology, University of Leipzig, Haertelstrasse 16-18, 04107 Leipzig, Germany; 2 LIFE - Leipzig Research Center for Civilization Diseases, University of Leipzig, Philipp-Rosenthal-Strasse 27, 04103 Leipzig, Germany

**Keywords:** Chemotherapy, Filgrastim, Granulopoiesis, Haematotoxicity, Leucopenia, Pegfilgrastim

## Abstract

**Background:**

The human granulocyte colony-stimulating factor (G-CSF) is routinely applied to support recovery of granulopoiesis during the course of cytotoxic chemotherapies. However, optimal use of the drug is largely unknown. We showed in the past that a biomathematical compartment model of human granulopoiesis can be used to make clinically relevant predictions regarding new, yet untested chemotherapy regimen. In the present paper, we aim to extend this model by a detailed pharmacokinetic and -dynamic modelling of two commonly used G-CSF derivatives Filgrastim and Pegfilgrastim.

**Results:**

Model equations are based on our physiological understanding of the drugs which are delayed absorption of G-CSF when applied to the subcutaneous tissue, dose-dependent bioavailability, unspecific first order elimination, specific elimination in dependence on granulocyte counts and reversible protein binding. Pharmacokinetic differences between Filgrastim and Pegfilgrastim were modelled as different parameter sets. Our former cell-kinetic model of granulopoiesis was essentially preserved, except for a few additional assumptions and simplifications. We assumed a delayed action of G-CSF on the bone marrow, a delayed action of chemotherapy and differences between Filgrastim and Pegfilgrastim with respect to stimulation potency of the bone marrow. Additionally, we incorporated a model of combined action of Pegfilgrastim and Filgrastim or endogenous G-CSF which interact via concurrent receptor binding. Unknown pharmacokinetic or cell-kinetic parameters were determined by fitting the predictions of the model to available datasets of G-CSF applications, chemotherapy applications or combinations of it. Data were either extracted from the literature or were received from cooperating clinical study groups. Model predictions fitted well to both, datasets used for parameter estimation and validation scenarios as well. A unique set of parameters was identified which is valid for all scenarios considered. Differences in pharmacokinetic parameter estimates between Filgrastim and Pegfilgrastim were biologically plausible throughout.

**Conclusion:**

We conclude that we established a comprehensive biomathematical model to explain the dynamics of granulopoiesis under chemotherapy and applications of two different G-CSF derivatives. We aim to apply the model to a large variety of chemotherapy regimen in the future in order to optimize corresponding G-CSF schedules or to individualize G-CSF treatment according to the granulotoxic risk of a patient.

## Introduction and background

### Background

The human granulocyte colony-stimulating factor (G-CSF) is routinely applied in various cancer chemotherapy regimen in order to ameliorate or prevent neutropenia caused by the unspecific toxicity of the drugs used [1-4]. G-CSF proved to be highly potent in stimulating granulopoiesis via several modes of action such as mitotic activation of granulopoietic progenitors and precursors, accelerated maturation of bone marrow cell stages and increased release of mature bone marrow cells [5-10].

In case of conventional (non-myeloablative) chemotherapies, the haematopoietic system usually recovers without further medication. But, G-CSF can significantly speed up this process allowing dose- and time-intensifications of multi-cycle chemotherapies [11,12]. While platelets and red blood cells can show cumulative toxicity during the course of intensified regimen, appropriate G-CSF prophylaxis often results in a complete recovery of circulating granulocytes within one therapy cycle, i.e. within two or three weeks [4,13].

Several pharmaceutical derivatives of G-CSF are available now. The first generation of G-CSF pharmaceuticals were recombinant derivatives such as Filgrastim (non-glycosylated) or Lenograstim (glycosylated). Both derivatives are virtually identical to endogenously produced G-CSF [14-16] but Filgrastim is more frequently used in clinical trials. Filgrastim is eliminated by both, renal elimination and specific degradation mediated by G-CSF receptors or neutrophil elastase [17-22]. This results in a short half-life *in vivo* requiring multiple injections during one cycle of chemotherapy. As next generation G-CSF derivative, Pegfilgrastim (pegylated Filgrastim) was developed in order to improve the pharmaceutical properties of Filgrastim. Indeed, Pegfilgrastim shows a remarkably prolonged half-life in vivo mainly due to reduced renal clearance [23-25]. Therefore, only one injection (with fixed dose) is required during one chemotherapy cycle. On the other hand, pegylation of proteins also might reduce the receptor binding affinity, and withit, the efficacy of the drug [26-29]. But for Pegfilgrastim, this effect appears to be less important than the gain in half-life, since it is generally believed that a single injection of Pegfilgrastim is at least as effective as multiple injections of Filgrastim in treating neutropenia [2,30-33]. There are some ongoing efforts to further improve the pharmacokinetic properties of G-CSF derivatives by additional pegylations (e.g. Maxy-G34, [29]). Although the application of pegylated G-CSF is much more convenient for both patients and clinicians, we believe that Filgrastim will not completely be replaced since it can be applied more individually e.g. in dependence on the neutropenic risk of a patient or in cases when granulopoietic recovery is insufficient while pegylated G-CSF was applied [34]. Additionally, for the purpose of stem cell mobilization, Filgrastim is not inferior compared to Pegfilgrastim but has less severe side-effects [35].

In view of the highly differing pharmacokinetic properties of the available G-CSF derivatives, we constructed pharmacokinetic models of the G-CSF derivatives Filgrastim, Pegfilgrastim and the novel Maxy-G34 in mice and rats [29,36]. Our aim was to identify basic pharmacokinetic model mechanisms especially with respect to the degradation of G-CSF *in vivo* and to compare the resulting pharmacokinetic parameters between the G-CSF derivatives. We now aim to translate these model insights to humans.

Effectiveness of G-CSF treatment depends on many variable therapy parameters such as applied chemotherapy, individual factors, G-CSF derivative used, and especially, its dosing and timing schedule [4,34,37,38]. Chemotherapy induced neutropenia and G-CSF induced granulocytosis via different modes of action in combination with a strong specific elimination of G-CSF mediated by circulating granulocytes results in complex dynamics of both G-CSF serum concentration and circulating granulocytes as well. In consequence, the optimization of G-CSF treatment is a non-trivial task and cannot be performed solely on the basis of clinical trials. We showed in the past that biomathematical cell-kinetic models of granulopoiesis under chemotherapy and G-CSF support are useful to optimize chemotherapy regimen regarding granulotoxicity [39-41]. Our former model already included a preliminary model of Filgrastim application. In the present paper, we update our model with respect to improved pharmacokinetic and dynamic modelling of Filgrastim and Pegfilgrastim based on our models in mice. Additionally, our cell-kinetic model has been improved by a more elaborated model of chemotherapy action. The resulting model is now able to explain the time courses of granulocytes and G-CSF serum concentrations for virtually all datasets published in the literature. We also discuss how the model can be used to optimize G-CSF scheduling of chemotherapies.

### Structure of the human model of granulopoiesis under chemotherapy with G-CSF support

**Figure 1:**
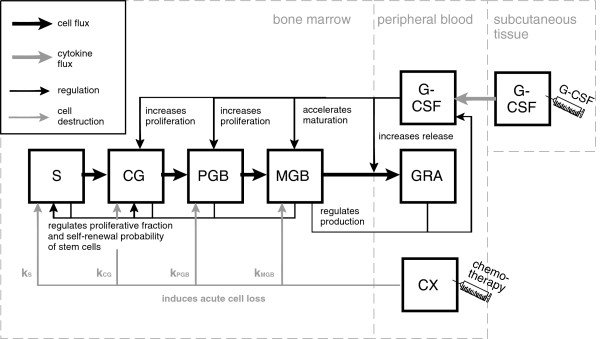
**(Basic structure of the cell-kinetic model of granulopoiesis).** Major model compartments describing granulopoietic cell stages are *S* (pluripotent stem cells), *CG* (colony forming units of granulocytes and macrophages), *PGB* (proliferating granulopoietic blasts), *MGB* (maturing granulopoietic blasts - subdivided into metamyelocytes (*G*4),banded granulocytes (*G*5) and segmented granulocytes (*G*6)) and *GRA* (circulating granulocytes). The system is regulated by feedback loops. A major loop is mediated by G-CSF which is produced endogenously but can also be applied subcutaneously. Chemotherapy (*CX*) induces acute cell loss. The model is essentially the same as in [40].

Our cell kinetic model of granulopoiesis is an ordinary differential equations system modelling the time-dependent content of and the fluxes between the following cell compartments: *S* (pluripotent stem cells), *CG* (colony forming units of granulocytes and macrophages), *PGB* (proliferating granulopoietic blasts), *MGB* (maturing granulopoietic blasts - subdivided into metamyelocytes (*G*4), banded granulocytes (*G*5) and segmented granulocytes (*G*6)) and *GRA* (circulating granulocytes). At this, the efflux of one compartment equals the influx of the subsequent compartment. The system is highly regulated via growth factor mediated feedback loops. The most important one is G-CSF which regulates the compartments *CG*, *PGB* and *MGB*, but not *S*[5-10,42,43]. Modes of action comprise improvement of proliferation, acceleration of maturation and improvement of the release of mature blood cells from bone marrow to blood. The latter one is also denoted as *postmitotic amplification* in the following. Production and consumption of G-CSF are regulated by mature cells. We also modelled a subcutaneous compartment in which G-CSF is usually injected. Chemotherapy induces an instantaneous depletion of bone marrow cell stages which is specific for each cell stage and dependent on the applied drugs and drug doses. The cell-kinetic model is essentially the same as presented and discussed in [40], except for a few changes which we will discuss later. Basic model structure is shown in Figure 1. A complete set of model equations and parameters is presented in the Additional file 1.

For unperturbed granulopoiesis, the model is autonomous and has a single fixed-point (steady-state). This fixed-point appears to be stable for the parameter set which we propose later, i.e. transient perturbations result in damped oscillations until the fixed-point is re-established. Permanent perturbations such as constant G-CSF stimulation or chemotherapy damage result in new fixed-points of increased or depressed granulopoiesis, respectively.

However, stable oscillations of the system can occur for alternative parameter sets especially if parameters of stem cell regulation are changed.

In general, we assume that the system is in steady-state at the beginning of any treatment. That is, initial values equals normal values.

### Basic model mechanisms

In this section, we briefly describe two important regulation mechanisms of our granulopoiesis model which are needed in the following. A more detailed discussion of all regulation mechanisms of the model is given in [40].

#### Z-function

Most of our cell kinetic parameters such as amplification or transition times are regulated between a minimum and a maximum value by a so called Z-function, which is a function of another quantity such as circulating G-CSF. We use the following class of Z-functions:

(1) $$\begin{array}{l} Y=Z\left(X\right)\\ \;=\left\{\begin{array}{l} Y^{\text{max}}-\left(Y^{\text{max}}-Y^{\text{min}}\right)e^{-\text{ln}\left(\frac{Y^{\text{max}}-Y^{\text{min}}}{Y^{\text{max}}-Y^{\text{nor}}}\right)X^{b_{Y}}}\\ \;\text{for}\;Y^{\text{min}}<Y^{\text{nor}}<Y^{\text{max}}\;\text{or}\;Y^{\text{min}}>Y^{\text{nor}}>Y^{\text{max}\;,}\\ Y^{\text{nor}}\;\text{for}\;Y^{\text{min}}=Y^{\text{nor}}=Y^{\text{max}} \end{array}\right) \end{array}$$

where *X* is the regulator of the quantity *Y * and *Y*^*min*^, *Y*^*nor*^, *Y*^*max*^, *b*_*Y *_are the parameters of the Z-function for the regulation of *Y *. The parameter *b*_*Y *_defines the steepness of the function and is called *sensitivity parameter* in the following.

#### Modelling of delays

The maturation of cells and the transition between maturing compartments is neither a random first order transition without dependence of cell age (which is equivalent to an exponential distribution of transit times) nor a transition with fixed time delay. In [40] we showed that a cascade of first order transitions results in a Gamma distribution of the transition times. More precisely, if we divide a compartment with transit time *T* in *N* subcompartments connected by first order transitions with time *T*/*N*, the resulting distribution is a Gamma distribution with expectation *T* and variance *T*^2^/*N*. Hence, the number of subcompartments corresponds to a variance estimate regarding the transition time.

The method of dividing compartments into subcompartments in order to introduce an element of time delay will later be adopted for the modelling of delayed G-CSF or chemotherapy actions.

## Refinement of the model

### Pharmacokinetic model assumptions

Our pharmacokinetic model of G-CSF is essentially based on models developed for mice and rats [29,36]. The underlying model assumptions are as follows: 

1. The pharmacokinetic model contains three compartments in which G-CSF is present: The subcutaneous compartment $C_{G-\mathrm{CSF}}^{\mathrm{sc}}$ in which G-CSF is usually injected, the central compartment $C_{G-\mathrm{CSF}}^{\mathrm{cent}}$ in which G-CSF is haematologically active and a peripheral compartment $C_{G-\mathrm{CSF}}^{\mathrm{per}}$ representing reversible bindings of G-CSF (e.g. protein binding [44]).

2. Subcutaneously injected G-CSF results in a delayed influx of G-CSF into the central compartment caused e.g. by lymphatic absorption [45]. The delay is modelled by the division of the subcutaneous compartment into two subcompartments.

3. Transitions between central and peripheral compartment are reversible and are modelled by a first order kinetic in both directions [46].

4. Endogenous G-CSF is produced by endothelial cells [47]. The production is regulated by the demand of mature granulocytes. We implemented a phenomenological rather than mechanistic modelling of this principle. At this, the production of endogenous G-CSF is modelled as a function of the content of the final bone marrow compartment and circulating granulocytes. This is in complete analogy to former versions of our model [40].

5. Since the bioavailability of G-CSF derivatives is dose-dependent [29], we assume that a part of the applied G-CSF is removed from the subcutaneous compartment without entering the central compartment. This is modelled by a Michaelis-Menten kinetic within the first subcompartment of the subcutaneous tissue.

6. G-CSF is irreversibly removed from the central compartment by two independent processes: An unspecific renal elimination which is modelled by a first order kinetic [24,46] and a specific degradation mediated by the number of circulating granulocytes. The latter one is modelled by a Michaelis-Menten kinetic which is assumed to be proportional to the number of circulating granulocytes [36,48-51]. Two key mechanisms are discussed for the specific degradation which are cleavage by neutrophil elastase [20,21,52,53] and G-CSF receptor binding and internalization [19,22,54-56]. Since neutrophil elastase is mainly produced by granulocytes [20], proportionality of degradation with granulocyte count can be assumed for the first mechanism. For the second mechanism, the proportionality assumption is less clear since the majority of G-CSF receptors are within the bone marrow which dynamics are somewhat different from the dynamics of mature granulocytes.

7. Differences in G-CSF derivatives are model by different model parameters rather than differences in model structure. In view of the high similarity of Filgrastim and endogenous G-CSF [14-16], we assume that the pharmacokinetic and -dynamic parameters of Filgrastim and endogenous G-CSF are the same. In contrast, we assume differences between Filgrastim and Pegfilgrastim for some of the parameters. More precisely, pharmacokinetic differences were assumed with respect to absorption, distribution and degradation of the G-CSF pharmaceuticals in order to model observed differences in G-CSF serum dynamics after subcutaneous applications [23-25]. Pharmacodynamik differences are modelled by different parameterizations of the G-CSF mediated regulatory mechanisms (Z-functions). The latter is motivated by experimental results suggesting a reduced receptor binding affinity of pegylated G-CSF [26-29].

**Figure 2:**
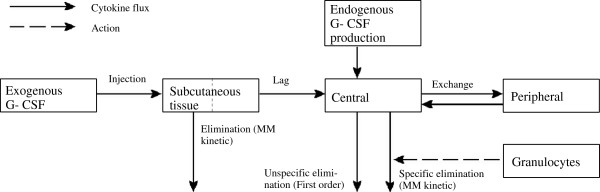
**(Model structure of the pharmacokinetic model of G-CSF).** The major compartments, cytokine fluxes and regulations are presented (MM = Michaelis Menten kinetic). The subcutaneous compartment is divided into two subcompartments with first order transition.

Based on these assumptions, we formulate the pharmacokinetic model equations in the next section. A schematic structure of the model can be found in Figure 2.

### Pharmacokinetic model equations

#### Endogenous production

According to [40], the relative G-CSF production $P_{G-\mathrm{CSF}}^{\mathrm{endo}}$ is a Z-function of the relative content of segmented granulocytes in bone marrow and granulocytes in circulation.

(2) $$P_{G-\text{CSF}}^{\text{endo}}=Z\left(\frac{\omega_{G6}C_{G6}+\omega_{\text{GRA}}C_{\text{GRA}}}{\omega_{G6}C_{G6}^{\text{nor}}+\omega_{\text{GRA}}C_{\text{GRA}}^{\text{nor}}}\right)$$

where *ω*_*G*6_ and *ω*_*GRA*_ are weighting parameters. It holds that $P_{G-\text{CSF}}^{\text{endo}\text{\_}\text{nor}}=1$ whereas all other parameters of the Z-functions are free parameters.

#### Exogenous G-CSF application

Exogenous G-CSF applications are modelled by an injection function. Let

$$\text{Hv}(t)=\left\{\begin{array}{c} 0\;\;:\;\;t\leq 0\\ 1\;\;:\;\;t>0 \end{array}\right)$$

be the Heaviside-function, then the injection function reads as follows:

(3) $$P_{G-\text{CSF}}^{\text{exo}}=\overset{L}{\underset{i=1}{\sum }}d_{G-\text{CSF}}\left(t_{i}\right)\frac{\text{Hv}\left(t-t_{i}\right)-\text{Hv}\left(t-t_{i}-t_{\text{inf}}\right)}{t_{\text{inf}}}$$

where *t*_*i *_≥ 0 (*i *= 1,…,*L*) are the time points of G-CSF injections and *d*_*G*−*CSF*_(*t*_*i*_) are the corresponding doses (in *μg*). The parameter *t*_*inf *_is the duration of the injection which we assumed to be constant (*t*_*inf *_= 5*s*).

The injection function is specific for each G-CSF derivative and site of injection (subcutaneous, intravenous). That is, for concurrent Filgrastim and Pegfilgrastim applications injections at concurrent sites one needs a maximum of four injection functions $P_{G-\text{CSF}}^{\text{exo}\_\text{sc}\_\text{fil}}$, $P_{G-\text{CSF}}^{\text{exo}\_\text{iv}\_\text{fil}}$ for subcutaneous and intravenous Filgrastim injections and $P_{G-\text{CSF}}^{\text{exo}\_\text{sc}\_\text{peg}}$ and $P_{G-\text{CSF}}^{\text{exo}\_\text{iv}\_\text{peg}}$ for subcutaneous and intravenous Pegfilgrastim injections respectively.

#### Subcutaneous compartment

The subcutaneous compartment is divided into two subcompartments *sc*_1 and *sc*_2 where the efflux of the first compartment is the influx to the second compartment. G-CSF is applied to the first subcompartment (second term of (4)). In the first subcompartment there is a dose-dependent loss of G-CSF modelled by a Michaelis-Menten kinetic (third term of (4)). For Filgrastim injections it holds that

(4) $$\begin{array}{ll} \frac{d}{\text{dt}}C_{G-\text{CSF}}^{\text{sc}\_1}=P_{G-\text{CSF}}^{\text{exo}\_\text{sc}\_\text{fil}}-k_{\text{sc}}^{F}C_{G-\text{CSF}}^{\text{sc}\_1}-\frac{v_{\text{max}}^{F}C_{G-\text{CSF}}^{\text{sc}\_1}}{k_{m}^{F}+C_{G-\text{CSF}}^{\text{sc}\_1}} & \; \end{array}$$

(5) $$\begin{array}{ll} \frac{d}{\text{dt}}C_{G-\text{CSF}}^{\text{sc}\_2}=k_{\text{sc}}^{F}\left(C_{G-\text{CSF}}^{\text{sc}\_1}-C_{G-\text{CSF}}^{\text{sc}\_2}\right) & \; \end{array}$$

with the initial values $C_{G-\text{CSF}}^{\text{sc}\_1}(0)\;\;=\;\;C_{G-\text{CSF}}^{\text{sc}\_2}(0)\;\;=\;\;0$. For Pegfilgrastim injections the first term of the right-hand side of (4) is substituted by $P_{G-\text{CSF}}^{\text{exo}\_\text{peg}\_\text{sc}}$. Likewise, the Filgrastim parameters $k_{\text{sc}}^{F}$, $v_{\text{max}}^{F}$ and $k_{m}^{F}$ are substituted by corresponding Pegfilgrastim parameters.

#### Central compartment

For Filgrastim injections it holds that

(6) $$\begin{array}{lll} \frac{d}{\text{dt}}C_{G-\text{CSF}}^{\text{cent}}\;\;=\;\; & P_{G-\text{CSF}}^{\text{ref}}P_{G-\text{CSF}}^{\text{endo}}+P_{G-\text{CSF}}^{\text{exo}\_\text{fil}\_\text{iv}}+k_{\text{sc}}^{F}C_{G-\text{CSF}}^{\text{sc}\_2}\;\\ & -k_{u}^{F}C_{G-\text{CSF}}^{\text{cent}}-k_{\text{cp}}^{F}C_{G-\text{CSF}}^{\text{cent}}+k_{\text{pc}}^{F}C_{G-\text{CSF}}^{\text{per}}\; & \;\\ & -\frac{v_{\text{max}}^{\text{GRA}\_F}C_{G-\text{CSF}}^{\text{cent}}}{k_{m}^{\text{GRA}\_F}+C_{G-\text{CSF}}^{\text{cent}}}C_{\text{GRA}}^{\text{rel}}.\; & \; \end{array}$$

The balance equation (6) contains terms in the following order: The endogenous production, a potential intravenous injection, the influx from the subcutaneous compartment, the unspecific elimination, the efflux to the peripheral compartment, the influx from the peripheral compartment and the specific elimination (Michaelis-Menten kinetic) which is proportional to the relative granulocyte concentration $C_{\text{GRA}}^{\text{rel}}$. The corresponding equation for Pegfilgrastim is the same except for the endogenous production, which is zero, the intravenous injection function which is substituted by $P_{G-\text{CSF}}^{\text{exo}\_\text{peg}\_\text{iv}}$, the parameters and the initial value which is again zero.

The relative endogenous production function of G-CSF is multiplied by a constant $P_{G-\text{CSF}}^{\text{ref}}$ in order to adjust the normal value of $C_{G-\text{CSF}}^{\text{cent}}$ to a reference amount of G-CSF which is the product of the distribution volume $V_{D}^{F}$ and the reference G-CSF serum concentration $C_{G-\text{CSF}}^{\text{cent}\_\text{ref}}$ which will be determined later on the basis of measurements available from the literature. Assume that $C_{G-\text{CSF}}^{\text{cent}}(0)=C_{G-\text{CSF}}^{\text{cent}\_\text{nor}}=V_{D}^{F}C_{G-\text{CSF}}^{\text{cent}\_\text{ref}}$, the parameter $P_{G-\text{CSF}}^{\text{ref}}$ can be calculated exploiting the steady-state conditions of equation (6), i.e. $P_{G-\text{CSF}}^{\text{endo}}(0)=1$, $P_{G-\text{CSF}}^{\text{exo}\_\text{fil}\_\text{iv}}(0)=0$ and $-k_{\text{cp}}^{F}C_{G-\text{CSF}}^{\text{cent}}(0)+k_{\text{pc}}^{F}C_{G-\text{CSF}}^{\text{per}}(0)=0$:

(7) $$P_{G-\text{CSF}}^{\text{ref}}=V_{D}^{F}C_{G-\text{CSF}}^{\text{cent}\_\text{ref}}\left(k_{u}^{F}+\frac{v_{\text{max}}^{\text{GRA}\_F}}{k_{m}^{\text{GRA}\_F}+V_{D}^{F}C_{G-\text{CSF}}^{\text{cent}\_\text{ref}}}\right)$$

#### Peripheral compartment

For both G-CSF derivatives, we have

(8) $$\begin{array}{ll} \frac{d}{\text{dt}}C_{G-\text{CSF}}^{\text{per}}=k_{\text{cp}}C_{G-\text{CSF}}^{\text{cent}}-k_{\text{pc}}C_{G-\text{CSF}}^{\text{per}} & \; \end{array}$$

(9) $$\begin{array}{ll} C_{G-\text{CSF}}^{\text{per}}(0)=C_{G-\text{CSF}}^{\text{per}\_\text{nor}}\;\;=\;\;V_{D}\frac{k_{\text{cp}}}{k_{\text{pc}}}C_{G-\text{CSF}}^{\text{cent}\_\text{ref}} & \; \end{array}$$

where the parameters *k*_*cp*_, *k*_*pc*_ and *V*_*D*_ are specific for Filgrastim and Pegfilgrastim respectively.

### New pharmacodynamic model assumptions

The pharmacodynamic model describes the dynamics of the bone marrow cell stages, circulating granulocytes, G-CSF, corresponding regulations and the action of chemotherapy as well. In Table 1 we present the major model compartments and its regulatory features. A complete set of equations can be found in the Additional file 1.

**Table 1:** Major compartments of the pharmacokinetic model and corresponding regulations

**Compartment**	**Regulations**	**Regulator**
*S*	proliferative fraction	bone marrow content
	self-renewal probability	bone marrow content
*CG*	proliferative fraction	bone marrow content
	amplification	G-CSF
	transition time	G-CSF
*PGB*	amplification	G-CSF
	transition time	G-CSF
*MGB*	post-mitotic amplification	G-CSF
	transition time	G-CSF
*GRA*	turn-over	-
*G-CSF*	endogenous production	late bone marrow cell stages
	specific degradation	*GRA*
	external applications	-

Most of these compartments are mediated by G-CSF. A complete set of model equations can be found in the Additional file 1.

We used the same pharmacodynamic model of G-CSF as presented in [40] except for a few simplifications and additional assumptions which we will discuss now: 

The cytokine GM-CSF (granulocyte macrophage colony-stimulating factor) was no longer considered in order to simplify the model. Since the endogenous productions of GM-CSF and G-CSF in our former model were both related to the demand of bone marrow cell stages, the time courses of endogenous G-CSF and GM-CSF after external perturbations are similar, making the cytokines undistinguishable from the modelling point of view (compare [40]). Therefore, only G-CSF was considered in the present model. It replaces GM-CSF regarding the regulation of the *CG* compartment. This simplification is also biologically plausible since G-CSF receptors are also expressed at myeloid progenitors [42].

The transition times *T*_*CG *_and *T*_*PGB *_were constant in our former version of the model but are now dependent on G-CSF via a Z-function. This model assumption was made in order to account for the increased number of mitoses in these compartments. But, due to the lack of data, it must be considered as speculative.

Since Pegfilgrastim and Filgrastim are supposed to have different G-CSF receptor binding affinities [26-29], we assume different regulatory Z-functions for both derivatives. But we assumed the same pharmacodynamic parameters for Filgrastim and endogenously produced G-CSF.

While in our former model version the G-CSF concentration instantaneously affected the value of the Z-functions, we now introduce a time delay regarding G-CSF action. This was motivated by transduction network analyses which revealed a delayed response of the transcriptome to G-CSF stimulations [57]. The delay is modelled by a cascade of four subcompartments (see section “Basic model mechanisms”). The efflux of the last subcompartment is the delayed G-CSF concentration $C_{G-\text{CSF}}^{\text{cent}\_\text{rel}\_\text{del}}$ which is the new argument of our Z-functions.

(10) $$\begin{array}{ll} \frac{d}{\text{dt}}C_{G-\text{CSF}}^{1}=C_{G-\text{CSF}}^{\text{cent}\_\text{rel}}-D_{G-\text{CSF}}\cdot C_{G-\text{CSF}}^{1} & \; \end{array}$$

(11) $$\begin{array}{ll} \frac{d}{\text{dt}}C_{G-\text{CSF}}^{i}=D_{G-\text{CSF}}\left(C_{G-\text{CSF}}^{i-1}-C_{G-\text{CSF}}^{i}\right)\;i=2,\ldots ,4 & \; \end{array}$$

with $C_{G-\text{CSF}}^{\text{cent}\_\text{rel}\_\text{del}}=D_{G-\text{CSF}}\cdot C_{G-\text{CSF}}^{4}$. The delay parameter *D*_*G*−*CSF *_is specific for Pegfilgrastim and Filgrastim but constant for all Z-functions. Hence, (1) reads

(12) $$Y=Z_{Y}\left(C_{G-\text{CSF}}^{\text{cent}\_\text{rel}\_\text{del}}\right)$$

for all quantities *Y* regulated by G-CSF. At this, the normal value of the Z-function *Y*^*nor *^refers to $C_{G-\text{CSF}}^{\text{nor}}$ for endogenous G-CSF or Filgrastim and to an absolute amount of 1*μ*g for Pegfilgrastim.

In case of Pegfilgrastim injections, Pegfilgrastim and endogenous G-CSF compete with respect to receptor binding. To model this process, the Z-functions of Pegfilgrastim and Filgrastim were added using a weighting factor *ω*_*P *_which is again a Z-function of the quotient of the two doses with minimum 0 and maximum 1.

(13) $$\begin{array}{lll} \omega_{P} & =Z_{\omega_{P}}\left(C_{G-\text{CSF}}^{\text{cent}\_\text{rel}\_\text{del}\_\text{peg}}/C_{G-\text{CSF}}^{\text{cent}\_\text{rel}\_\text{del}\_\text{fil}}\right)\;\\ & \;\;\;\;\;\;\;\;\;\;\;\text{with}\;\omega_{P}^{\text{min}}=0\;\text{and}\;\omega_{P}^{\text{max}}=1\; & \; \end{array}$$

(14) $$\begin{array}{ll} Z_{Y} & =\omega_{P}\cdot Z_{Y}\left(C_{G-\text{CSF}}^{\text{cent}\_\text{rel}\_\text{del}\_\text{peg}}\right)+\left(1-\omega_{P}\right)\cdot Z_{Y}\left(C_{G-\text{CSF}}^{\text{cent}\_\text{rel}\_\text{del}\_\text{fil}}\right)\; \end{array}$$

where *Y* is an arbitrary regulated quantity such as a transition time or an amplification. For all these quantities we assumed the same Z-function of the weighting parameter *ω*_*P*_.

In the previous version of our model, chemotherapy was modelled by an instantaneous depletion of bone marrow cells lasting exactly one day. Since independence of cytotoxic action of the single chemotherapy components was assumed, the effect of chemotherapy could be modelled by a step function with a step width of one day. Although the metabolism of cytotoxic drugs is usually fast, the nadir of bone marrow cell stages is typically reached a few days after the application [58]. To account for this observation, we delayed the toxic effect of chemotherapy applications by a cascade of four subcompartments in complete analogy to (10), (11) resulting in a delayed toxicity function *Ψ*^*GRA*^. The delay parameter is specific for the cytotoxic drugs used. Hence, two parameters are required to define the toxic effect of a chemotherapeutic drug to a single cell line: a delay parameter and a scaling factor of the toxicity function. While the delay is only specific for the applied chemotherapeutic drug but constant for all cell stages, the scaling factor is specific for both.

Since our model is a model of granulopoiesis, we can only make predictions for absolute neutrophil counts. However, in clinical practice often only leukocytes are available. In our former model version, we assumed proportionality of leukocytes and absolute neutrophil count which is only roughly correct [38,40]. To be more precise, we now calculate the leukocyte count as the sum of lymphocytes and granulocytes. To avoid a full model of lymphopoiesis, we modelled the reduced lymphocyte count under chemotherapy by an exponential function of the corresponding toxicity function.

(15) $$C_{\text{WBC}}(t)\;\approx \;c_{\text{LY}}\text{exp}\left(-\Psi^{\text{LY}}(t)\right)+c_{\text{GRA}}\frac{C_{\text{GRA}}(t)}{C_{\text{GRA}}^{\text{nor}}}$$

where *c*_*LY *_= 3000 cells per *μl *and *c*_*GRA *_= 4000 cells per *μl *are the normal concentrations of lymphocytes and granulocytes respectively. *Ψ*_*LY *_is the toxicity function for lymphocytes which is analogously defined as the toxicity functions of granulopoiesis (see 6.)

### Construction of toxicity functions

Since the precise structure of the toxicity function depends on the schedule of the chemotherapy, we demonstrate the construction of the toxicity functions using six cycles of CHOP therapy with a cycle duration of 14 days (6xCHOP-14) as example. During CHOP-14 therapy, the cytotoxic drugs cyclophosphamide, doxorubicine and vincristine are applied concomitantly at the first day of each cycle. Since contributions of these drugs to granulotoxicity cannot be separated, we assume a unique toxicity function for this drug combination. Hence, the chemotherapy injection function $\Psi_{6\times \text{CHOP}-14}^{\text{inj}}$ reads as follows:

$$\Psi_{6\times \text{CHOP}-14}^{\text{inj}}\;\;=\;\;\overset{6}{\underset{i=1}{\sum }}\frac{\text{Hv}\left(t-14d\cdot \left(i-1\right)\right)-\text{Hv}\left(t-14d\cdot \left(i-1\right)-t_{6\times \text{CHOP}-14}^{\text{inf}}\right)}{t_{6\times \text{CHOP}-14}^{\text{inf}}}$$

which is in analogy to (3). Here, no dose parameter is required since intensity of damage is defined by the toxicity parameters later. We set $t_{6\times \text{CHOP}-14}^{\text{inf}}=1d$. The delayed action of chemotherapy can now be modelled in analogy to (10), (11):

$$\begin{array}{lll} \frac{d}{\text{dt}}\Psi_{6\times \text{CHOP}-14}^{X\_1} & =\Psi_{6\times \text{CHOP}-14}^{\text{inj}}-D_{6\times \text{CHOP}-14}^{X}\cdot \Psi_{6\times \text{CHOP}-14}^{X\_1}\; & \;\\ \frac{d}{\text{dt}}\Psi_{6\times \text{CHOP}-14}^{X\_i} & =D_{6\times \text{CHOP}-14}^{X}\cdot \left(\Psi_{6\times \text{CHOP}-14}^{X\_(i-1)}-\Psi_{6\times \text{CHOP}-14}^{X\_i}\right)\; & \; \end{array}$$

where *i *= 2,…,4, *X*∈{*LY*,*GRA*}, i.e. the delay parameter *D* is specific for each chemotherapy and different for the toxicity functions of granulocytes and lymphocytes. Now, the toxicity functions are defined as:

$$\begin{array}{lll} \Psi_{6\times \text{CHOP}-14}^{\text{LY}} & =k_{\text{LY}}\cdot D_{6\times \text{CHOP}-14}^{\text{LY}}\cdot \Psi_{6\times \text{CHOP}-14}^{\text{LY}\_4}\; & \;\\ \Psi_{6\times \text{CHOP}-14}^{\text{GRA}\_Y} & =k_{Y}\cdot D_{6\times \text{CHOP}-14}^{\text{GRA}}\cdot \Psi_{6\times \text{CHOP}-14}^{\text{GRA}\_4}\; & \; \end{array}$$

where *k* are the toxicity parameters and *Yε*{*S*,*CG*,*PGB*,*MGB*}, i.e. the toxicity function is specific for the different cell stages of granulopoiesis. If drugs are applied at different schedules, corresponding toxicity functions are added. The sketched principle can easily be generalized to derive toxicity functions of arbitrary chemotherapy schedules.

## Model calibration, parameter estimation and validation

### Estimation of parameters

A main goal of our study is to construct pharmacokinetic models of Filgrastim and Pegfilgrastim. Based on the pharmacokinetic model equations presented above, a set of unknown pharmacokinetic parameters needs to be determined.

Since detailed bone marrow data of human granulopoiesis are not available, most of the bone marrow parameters are only known up to a certain range or are completely unknown. Furthermore, model parameters regarding sensitivity of regulatory mechanisms (sensitivity parameters) have no direct biological measurable equivalent. Finally, we want to apply the model to chemotherapy settings requiring a quantification of corresponding toxicity and delay parameters. Many of the parameters of the present model version were adopted from an earlier version of the model especially if they are not very sensitive regarding model behaviour (compare [40]). But the inclusion of new regulatory mechanisms (see section “New pharmacodynamic model assumptions”) made some adaptations of model parameters necessary.

To address these challenges, we established the following stepwise fitting procedure keeping parameters identified at the previous step constant. 

1. Pharmacokinetic model parameters were determined on the basis of available cytokine dynamics after G-CSF application. In order to model the specific elimination mechanism, we imprinted the corresponding data of granulocyte dynamics at this stage of modelling. We obtained a unique parameter set which is valid for all dosing and timing schedules of all scenarios considered.

2. Pharmacodynamic parameters were determined by fitting the predictions of the model to available granulocyte and leukocyte dynamics of different scenarios (G-CSF application and simple chemotherapies for which the number of chemotherapy parameters to be fitted is relatively low). The resulting parameter set is valid for all scenarios with and without chemotherapy applications. Since the stem cell compartment is the basis of all of our models of haematopoietic lineages, we decided to keep corresponding parameters constant and as presented in [40,59].

3. Afterwards, toxicity parameters of more complex chemotherapies can be estimated. More details of chemotherapy modelling, parameter estimation and exploration of patients with different risk of haematotoxicity can be found in a separate publication of our group (Wetzler et al., to appear).

A complete parameter list for our model is provided in the Additional file 1. Not all data sets were used to fit parameters. A few data sets were kept in reserve in order to validate the model.

### Available data sets

Data sets were collected from the literature by an extensive search. For our modelling purposes, close-meshed time series of G-CSF and absolute neutrophil counts (ANC) or leukocytes are especially valuable. Corresponding data were extracted from the publications as precise as possible using automated tools. Data sets for which no means or medians of the patients could be retrieved were neglected. Data sets comprise single or multiple applications of G-CSF in healthy volunteers and conventional chemotherapies of different diseases with or without G-CSF prophylaxis.

Additionally, we can rely on own clinical trials data for which one of us (Markus Loeffler) is the responsible biostatistician or for which we have cooperation agreements. Leukocyte raw data under chemotherapy are available from published studies of the *German High Grade Non-Hodgkin-Lymphoma Study Group*. These studies were conducted in accordance with the Declaration of Helsinki. Corresponding protocols were approved by the ethics review committee of each participating center. Written informed consent was obtained from the patient for publication of this report and any accompanying images. An overview of data used for model fitting and validation is given in Table 2.

**Table 2:** Data sets utilized for the establishment and validation of the granulopoiesis model

**Type of data**	**Disease**	**G-CSF schedules**	**Chemotherapy**	**References**
Phase I studies with Filgrastim	none	single application 3, 5, 10 *μ*g/kg	none	[31]
	none	single application 5, 10 *μ*g/kg	none	[60]
	none	10 applications 75, 150, 300, 600 *μ*g	none	[61]
	none	14 applications 30, 300 *μ*g	none	[62]
	none	single application 4, 8 *μ*g/kg	none	[63]
Phase I studies with Pegfilgrastim	none	30, 60, 100, 300 *μ*g/kg	none	[50]
Phase II studies with Pegfilgrastim	LuCa	30, 100, 300 *μ*g/kg	none	[64]
	NHL	6000 *μ*g, day 2	CHOP	[65]
Phase III studies with CX and w/wo Filgrastim	NHL	no G-CSF	CHOP-21*	[12]
	NHL	480 *μ*g, day 4–13	CHOP-14*	[12]
	NHL	480 *μ*g, day 6–12	CHOP-14*	[66]
Phase III studies with CX + Peg	NHL	6000 *μ*g, day 2, 4	CHOP-14*	[67]

Studies with access to raw data are indicated with an asterisk. CX = chemotherapy, LuCa = lung cancer, NHL = high-grade non-Hodgkin’s lymphoma.

### Fitting procedure

As mentioned above, unknown parameters of the model were determined by fitting the predictions of the model to available clinical data minimizing the *L*^1^ distance between logarithmized model and logarithmized median of data. More precisely, we have

(16) $$\int_{t_{0}}^{t_{1}}|\text{log}(f_{\text{model}}(t,k))-\text{log}f_{\text{data}}(t)|\text{dt}\to \underset{k}{\text{min}},$$

where *f*_*model*_(*t*,**k**) is the solution of the model equation system for the granulocyte compartment at the time *t* based on the parameter set **k **=* k*_1_,…*k*_*n*_. For each scenario, *t*_0_ and *t*_1_ describe the first and the last time point for which data are available. To obtain the curve log*f*_*data*_(*t*), the logarithms of the patients medians were linearly interpolated. Logarithms of data were used to provide an optimal fit of the nadir phase of cell counts. In the following, the left hand side of equation (16) is referred to as the *fitness function*.

As in our previous papers, evolutionary strategies were used for the numerical solution of the optimisation problem. This method is especially suitable for our problem since it requires a minimum of computationally expensive calculations of the fitness function, it can deal with a large number of free parameters with only a linear growth in effort and it is the only chance to obtain a global optimum as good as possible.

Evolutionary strategies are non-deterministic optimization methods which are based on the principles of evolution (mutation by chance, reproduction, realization of phenotypes and survival of the fittest). For mathematical optimisation, parameter settings were taken instead of livings, that is parental parameter settings are changed by chance (mutation), combined to form new parameter settings (reproduction) and were used to solve the model equation system (realization). The parameter settings for which one obtains a good agreement between the model prediction and the data were taken to create the next generation of parameter settings (survival of the fittest). The fitness function is a measure for this agreement. We used a (1+3) evolutionary strategy with self-adapting mutation step size most of the time. That is, one possibly immortal parent creates three children in each step. See also [68,69] for further details of evolutionary strategies.

Fitting of chemotherapy schedules requires additional parameters with respect to the chemotherapy toxicity. The delay parameter of the toxicity is specific for each drug but is the same for all bone marrow cell stages. Four toxicity parameters are required to model the cell stage specific toxicities. Another parameter represents increased toxicity for the first chemotherapy application (first cycle effect). Finally, two parameters represent the toxicity to the lymphopoetic system (eq. (15)). In general, modelling a chemotherapy required several sets of these parameters in order to model all drugs or drug combination with different schedules. However, for the purpose of model calibration, we only considered simple chemotherapy schedules in order to reduce the number of additional unknown parameters to be fitted. The CHOP regimens are based on the application of three cytotoxic drugs (cyclophosphamide, doxorubicine and vincristine) at the same time. Hence, only one set of chemotherapy parameters was assumed to model the effects of this drug combination. Additionally, for these regimens different G-CSF schedules are available, which is especially useful for the calibration of our pharmacokinetic and -dynamic models of Filgrastim and Pegfilgrastim.

Not all of our data sets were utilized for parameter fitting. A subset of data sets was kept in reserve in order to validate the model. Data sets used for model validation comprise the data of [63,65].

### Sensitivity analysis

Since our model contains several parameters which are speculative or unknown only up to a certain range, we peformed an extensive sensitivity analysis of all parameters. For this purpose, parameters were increased or decreased by 2.5% and the corresponding change of the fitness function was determined. At this, only affected model scenarios were considered. The changes of the fitness function were plotted as bar diagrams for each parameter in order to facilitate comparisons. Figures are shown in the Additional file 1.

### Simulation and numerical methods

Our model has been programmed with MATLAB 7.5.0.342 (R2007b) and SIMULINK toolbox (The MathWorks Inc., Natick, MA, USA). Simulations of the model were performed by numerical integration of the equation system using the variable step solver from Adams and Bashford implemented in the SIMULINK toolbox.

## Results

### Pharmacokinetic model of Filgrastim and Pegfilgrastim

Given the model equations of section “Pharmacokinetic model equations”, we determined the pharmacokinetic parameters for Filgrastim and Pegfilgrastim separately by fitting the model to time series of G-CSF serum concentrations after single or multiple application of one of the two G-CSF derivatives. Parameter estimates are shown in Table 3.

**Table 3:** Pharmacokinetic parameters of Filgrastim and Pegfilgrastim

**Parameter**	**Meaning**	**Filgrastim**	**Pegfilgrastim**
*k*_*sc*_	subcutaneous absorption [*h*^−1^]	0.161	0.107
*k*_*m*_	Michaelis-Menten constant of	34.7	5.5
	subcutaneous elimination [*μg*]		
*v*_*max*_	Maximum of subcutaneous	67.3	16.5
	elimination [*h*^−1^]		
*k*_*u*_	unspecific elimination [*h*^−1^]	0.441	0.087
$k_{m}^{\text{GRA}}$	Michaelis-Menten constant of	22.4	30.8
	specific elimination [*μg*]		
$v_{\text{max}}^{\text{GRA}}$	Maximum of specific elimination [*h*^−1^]	4.77	5.16
*k*_*cp*_	transition central to peripheral [*h*^−1^]	0.000	0.075
*k*_*pc*_	transition peripheral to central [*h*^−1^]	-	0.548
*V*_*D*_	distribution volume [*l*]	1.156	4.091
$C_{G-\text{CSF}}^{\text{cent}\_\text{ref}}$	reference G-CSF serum concentration $\left[\frac{\mu g}{l}\right]$	0.02	

**Figure 3:**
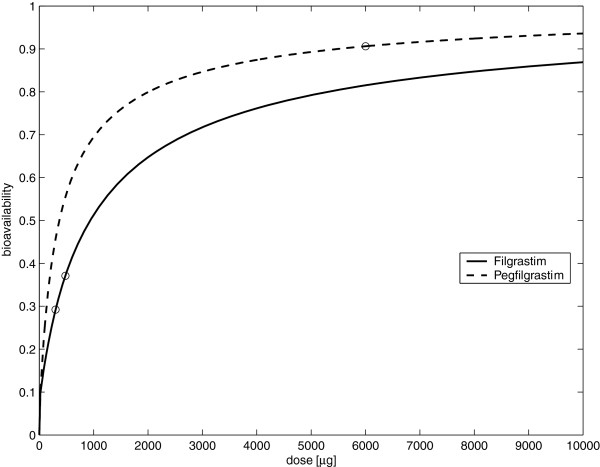
**(Estimated bioavailability of subcutaneously injected Filgrastim or Pegfilgrastim based on systematic model simulations): Bioavailability was estimated by calculating G-CSF amounts absorbed by the central compartment relative and the total amount of subcutaneously injected G-CSF (x-axis).** Due to the modelled loss in the subcutaneous tissue, the bioavailability is dose-dependent. Circles indicate estimates for pharmaceutically available doses of 300*μg *and 480*μg *of Filgrastim or 6000 *μg *of Pegfilgrastim respectively [12,67].

Compared to Pegfilgrastim, we estimated that Filgrastim is more easily absorbed from the subcutaneous compartment, has a lower bioavailability (see Figure 3) and a higher specific and unspecific elimination. Reversible binding is neglectable for Filgrastim but not for Pegfilgrastim. The distribution volume is higher for Pegfilgrastim than for Filgrastim.

**Figure 4:**
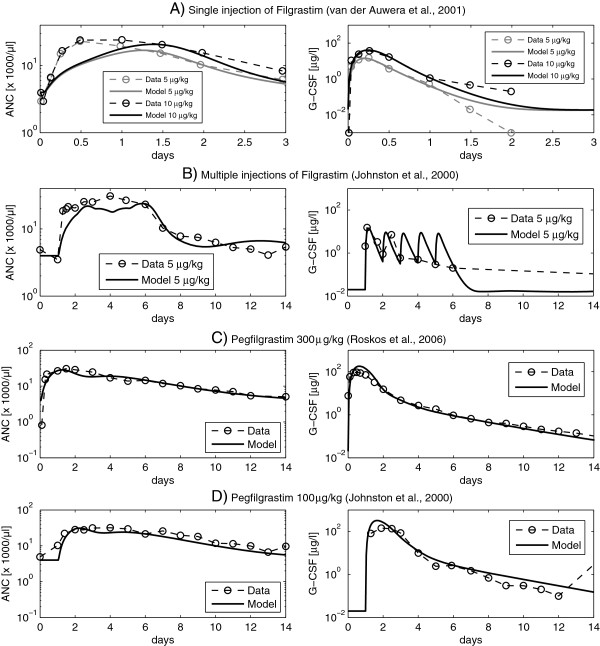
**(Comparison of model and data for G-CSF applications): Comparison of model and selected datasets of single Filgrastim injections (scenario A), multiple Filgrastim injections (scenario B) and single Pegfilgrastim injections of different doses (scenarios C and D).** For each scenario, we present the time courses of ANC and G-CSF, respectively. A complete list of scenarios can be found in the Additional file 1.

Combined with our pharmacodynamic model (see section “The new pharmacodynamic model”), these parameter estimates resulted in a good fit of all model scenarios. Examples are presented in Figure 4. A complete list of all scenarios is presented in the Additional file 1.

### The new pharmacodynamic model

Unknown pharmacodynamic parameters were determined by fitting the predictions of our model to available time courses of ANC or leukocytes after single or multiple injections of G-CSF or chemotherapy. All scenarios presented in section “Available data sets” were used for this simultaneous fitting process except for those reserved for model validation (compare section “Fitting procedure”). Fitted parameters resulted in a good fit of all model scenarios except for time points shortly after G-CSF injections. Examples are shown in Figure 4. All other scenarios of G-CSF application can be found in the Additional file 1. Chemotherapy scenarios are presented in the next section.

The most sensitive pharmacodynamic parameters influencing the behaviour of the model are parameters regarding the CG compartment (regulation of the proliferative fraction, transition time and amplification), the amplification in the PGB compartment and the postmitotic amplification. Sensitivity parameters of the regulation functions are generally less sensitive except for the sensitivity parameter of the regulation of the postmitotic amplification. A complete list of the results of the sensitivity analysis can be found in the Additional file 1.

**Figure 5:**
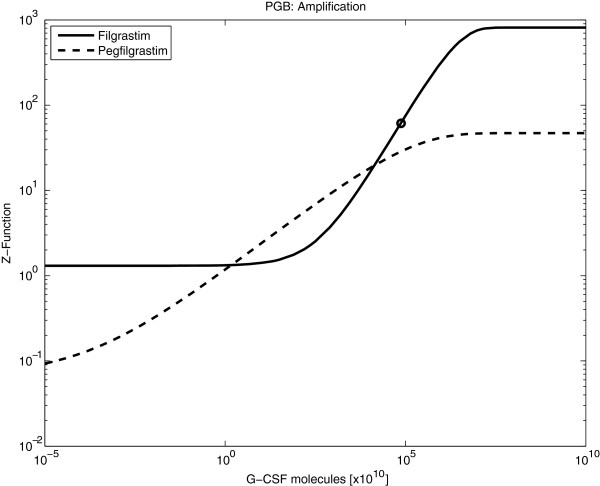
**(Regulation functions of Filgrastim and Pegfilgrastim): Comparison of Filgrastim and Pegfilgrastim with respect to the regulation function of the amplification in PGB.** The circle marks the value under steady-state conditions.

Pharmacodynamic differences between Filgrastim and Pegfilgrastim can be traced back to differences of the regulation functions. In general, compared to Pegfilgrastim, the regulation functions of Filgrastim express a higher sensitivity regarding changes of the G-CSF concentration, that is, greater slopes and higher values under maximum stimulation (see Figure 5 for an example).

**Figure 6:**
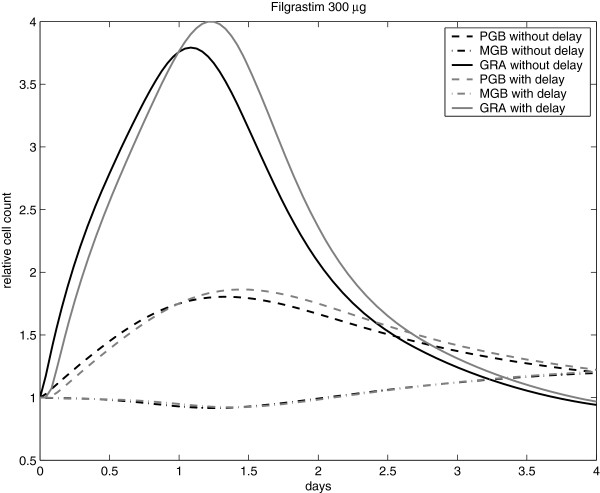
(Effect of G-CSF delay): Effect of the delay parameter of G-CSF action on cell-counts of specific cell compartments.

In contrast to our former model of granulopoiesis, we assumed a delayed effect of G-CSF (assumption 4 in section “New pharmacodynamic model assumptions”). Consequences of this assumption are studied in Figure 6 on the basis of model simulations of single Filgrastim injections.

The estimated delay is moderate in size resulting in a small shift of the time course of cell stages. This shift is negligible for *MGB* but more pronounced for the granulocyte compartment which can be explained by the postmitotic amplification mechanism (see [40]).

### The new chemotherapy model

**Figure 7:**
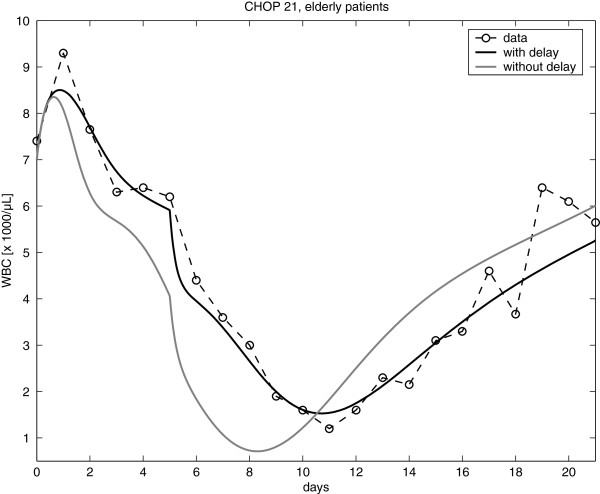
(Effect of chemotherapy delay): Effect of the delay parameter of the chemotherapy studied for the CHOP regimen.

Chemotherapy was modelled by a transient depletion of bone marrow cell stages. In contrast to our former model of granulopoiesis, we assumed a delay of the bone marrow depletion. The effect of this delay is studied in Figure 7.

The delay resulted in a later occurrence and a reduced depth of the nadir of leukocytes. The delay was assumed to be different for different chemotherapeutic drugs or drug combinations (see Wetzler *et al.* for further details).

**Figure 8:**
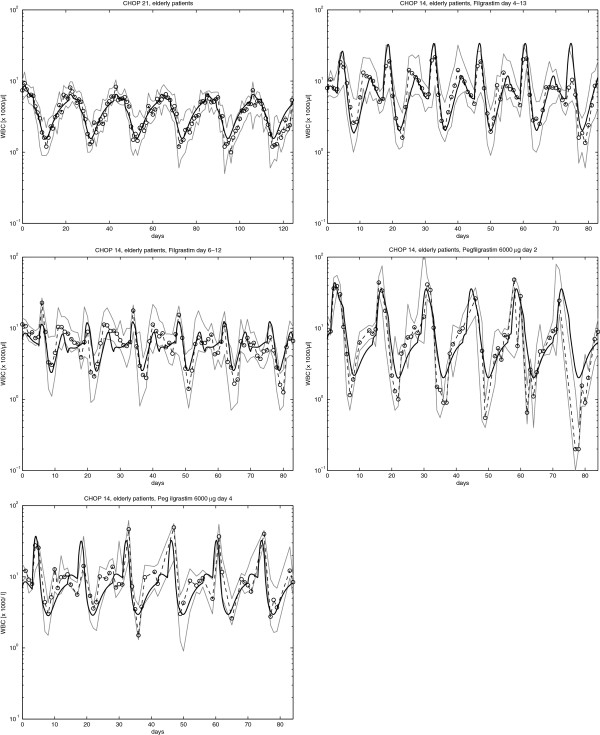
**(Comparison of model and data for chemotherapy scenarios): Comparison of model and data for the CHOP-21 regimen and time intensified CHOP-14 regimen supported by various Filgrastim and Pegfilgrastim schedules.** The solid line is the model prediction. Dots are patient medians at corresponding time points and the grey lines mark the interquartile range of the data. All scenarios are based on the same model parameters.

Data of the CHOP regimen were utilized to fit both, the pharmacokinetic and -dynamic model and the set of specific toxicity parameters as well. This set of toxicity parameters was valid for all G-CSF schedules applied as supportive therapy for CHOP. Results of these scenarios are shown in Figure 8.

Comparison of model and data revealed a good agreement. For almost all time points, the model curve is within the interquartile range of the data. Chemotherapy and delay parameters for the CHOP regimen can be found in the Additional file 1.

**Figure 9:**
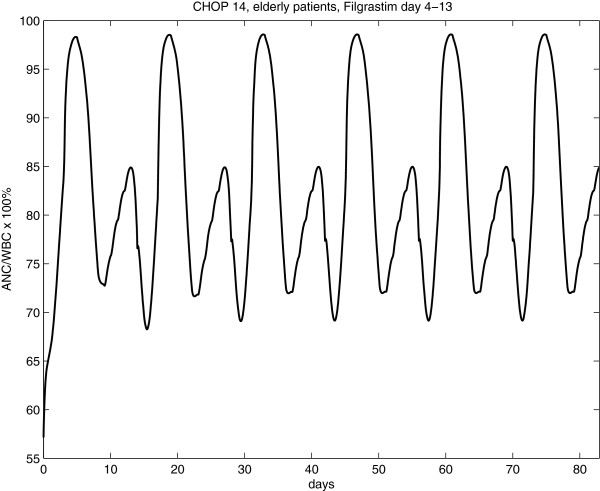
(Ratio of granulocytes and leukocytes): Based on model simulations, the ratio of granulocytes and leukocytes under CHOP-14 chemotherapy is predicted.

Since we assumed a simplified model of lymphocyte toxicity under chemotherapy, we can estimate the ratio of granulocytes and leukocytes under therapy, offering a possibility to validate the model. Results of the CHOP-14 regimen with Filgrastim application at day 4 to 13 are displayed in Figure 9. This ratio was estimated to be clearly not constant varying between 68% and 98%.

### Validation of the model

**Figure 10:**
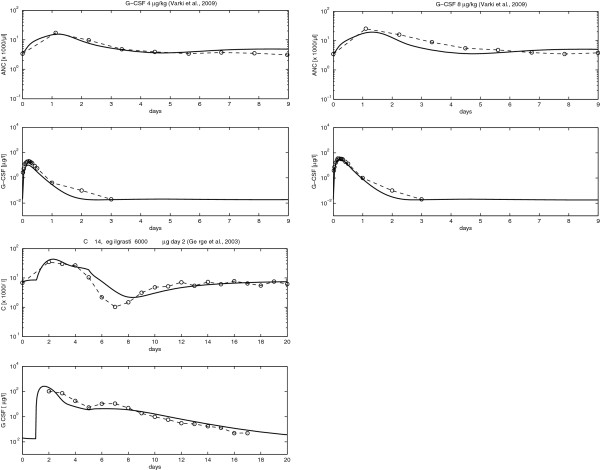
**(Validation of model): Validation of the model on the basis of two datasets not used for model fitting.** Solid line is the model prediction. Dots and dotted lines are the data and the interpolated data respectively.

A few datasets were kept in reserve in order to validate our model. The phase 1 data of Varki *et al.*[63] were not used for model fitting as well as the data of CHOP chemotherapy under Pegfilgrastim treatment of George *et al.*[65]. Compared to the CHOP data used for model fitting, the data of George *et al.*[65] comprise G-CSF serum levels as well. Both scenarios fit well with our model prediction (see Figure 10). No additional parameter fittings were performed to model these scenarios.

## Discussion

In the present paper, we developed an ordinary differential equations model of human granulopoiesis under chemotherapy and G-CSF support. The model was built on the basis of a former model of granulopoiesis of our group which now has been improved primarily by the incorporation of a detailed pharmacokinetic and -dynamic model of two G-CSF derivatives (Filgrastim and Pegfilgrastim). At this, the pharmacokinetic model was adopted from similar models developed for G-CSF applications in mice and rats developed by our group. Unknown model parameters were obtained by fitting the predictions of the model to available datasets. The combined pharmacokinetic and -dynamic model correctly predicts the time course of a variety of datasets comprising single or multiple injections of G-CSF into healthy volunteers or patients under CHOP chemotherapy. We were able to describe the differences between the G-CSF derivatives by a set of different pharmacokinetic- and -dynamic parameters. The model was validated on the basis of datasets not used for model fitting.

The presented model is by far not the first attempt to model granulopoiesis or G-CSF applications. Published models comprise for example pure pharmacokinetic models [25,46], pharmacokinetic and -dynamic models of G-CSF application on the cellular level [49], in healthy volunteers [50,51,70], for the treatment of cyclic neutropenia [71], for high-dose chemotherapy with stem cell transplantation [72,73] or for conventional chemotherapy patients [74,75]. We developed a model of human granulopoiesis under chemotherapy in the past including a preliminary model of Filgrastim application [40]. To the best of our knowledge, there is so far no granulopoiesis model of humans under conventional chemotherapy comprising a detailed pharmakokinetic- and dynamic model of the two G-CSF derivatives Filgrastim and Pegfilgrastim. This combination allows us to derive clinically meaningful applications of the model.

As mentioned, the presented model was based on a former model of our group. This model was based on biologically plausible assumptions regarding the production of mature granulocytes via a cascade of bone marrow cell stages, the action of chemotherapy and the action of growth factor mediated feedback loops. Equations describe the fluxes between cell compartments. G-CSF was modelled as the major regulatory element of both, the transition time and the amplification within the compartments. Chemotherapy was modelled by an instantaneous and transient cell loss of all bone marrow cell stages. Since these basic model assumptions were intensively discussed in our former paper [40] we will focus on our new model assumptions and parameters in the following.

The major improvement of our model is the incorporation of a detailed pharmacokinetic model of the two G-CSF derivatives Filgrastim and Pegfilgrastim. Both are widely used in clinical practice in order to ameliorate leukopoenia during cancer chemotherapy. The pharmacokinetic model was constructed in complete analogy to the pharmacokinetic models which we developed for mice and rats recently [29,36]. That is, we made the same physiological assumptions and used the same model equations but different parameters. Furthermore, we used the same model equations for both drugs assuming that pharmacokinetic differences between the drugs can be traced back to different parameters rather than different mechanisms of action.

The drugs were typically injected into the subcutaneous tissue resulting in a delayed absorption by the circulating blood compartment probably via lymphatic absorption [45]. The delay was modelled by a set of concatenated first-order differential equations rather than a fixed time delay. We showed in the past that this kind of modelling is equivalent to a Gamma-distributed transition time, which is biologically plausible. At this, the variance is determined by the number of subcompartments [40]. However, this variance appears to be of lesser importance for the model behaviour. In analogy to [36], we observed that a number of subcompartments between two and ten would also work well. To reduce the computational in the present model, we used the smaller number.

Data collected in mouse and rat experiments suggested that subcutaneously injected G-CSF has a dose-dependent bioavailability [29,36]. Therefore, we introduced a loss term into the equations of the subcutaneous compartment. Since there is some evidence of reversible protein binding of G-CSF molecules, we modelled a first order transition between the blood compartment and a peripheral compartment [46].

Our model assumptions regarding endogenous production of G-CSF are speculative. We assumed that the production is regulated between a minimum and a maximum value in dependence on bone marrow cellularity [47]. In steady-state, the production is constant in order to sustain a fixed serum concentration determined by averaged data from the literature.

Degradation of G-CSF was modelled by two independent processes, an unspecific renal clearance modelled by a first order transition and a specific degradation via neutrophil elastase or G-CSF receptors. All three degradation mechanisms are biologically well understood but their relative importance is unknown (see discussed literature in section “Pharmacokinetic model assumptions”). The specific degradation was modelled by a Michaelis-Menten kinetic which was assumed to be proportional to the number of granulocytes. For the degradation mechanism due to neutrophil elastase, this assumption seems to be appropriate [21]. On the other hand, since G-CSF receptors are also present in bone marrow progenitors and precursors [42], it appears to be less appropriate for the receptor-mediated clearance mechanism. Nevertheless, we assumed proportionality as the most parsimonious model resulting in a good agreement of predictions and data. The assumption also worked well for the pharmacokinetic modelling in mice [36] but not for rats [29]. Moreover, we experimented with alternative model assumptions assuming consumption of G-CSF by both, mature neutrophils and precursors which did not significantly improve the quality of our model predictions.

Variable numbers of G-CSF receptors per cell were observed in dependence on the G-CSF level [56]. Modelling this obervation would require additional assumptions and parameters. We decided to skip this in the current version of the model in view of the relatively good quality of model predictions in the clinical scenarios considered so far.

The model equations worked well to explain the dynamics of G-CSF serum concentrations after single or multiple injections of Filgrastim or Pegfilgrastim in healthy or diseased people. The same equations worked also well for a third G-CSF derivative, namely Maxy-G34, which is a novel G-CSF derivative currently under development by Maxygen Inc. However, due to a confidentiality agreement with Maxygen Inc., the results are not shown in the present paper.

In order to make predictions regarding the response of granulopoiesis after the application of G-CSF, it was necessary to attach a pharmacodynamic model of G-CSF applications. Since there is some evidence that the pegylations of the Pegfilgrastim molecule interact with its binding affinity to the G-CSF receptor [26-29], we decided to assume different regulation functions for Filgrastim and Pegfilgrastim. Hence, Z-functions of the transition times and amplifications in *CG*, *PGB* and *MGB* are assumed to be different for Filgrastim and Pegfilgrastim. On the other hand, we assumed the same Z-functions for Filgrastim and endogenous G-CSF. Due to the fact that Filgrastim/endogenous G-CSF and Pegfilgrastim were assumed to be different regarding Z-functions, it was necessary to merge the superimposing effects of Pegfilgrastim and endogenous G-CSF or concurrent Pegfilgrastim and Filgrastim applications as well. This was solved by a weighting factor which is regulated between zero and one in dependence on the ratio of Pegfilgrastim and Filgrastim or endogenous G-CSF in the system. If the Pegfilgrastim concentration is high or low, then the system is mainly influenced by the Z-function of Pegfilgrastim or Filgrastim respectively. Although this assumption is plausible, the complete regulation mechanism via the combined Z-functions must be considered as speculative, since especially the shape of the regulation functions can hardly be observed or measured.

Another speculative mechanism introduced into our model update was the delayed effect of G-CSF action. By model fitting, we estimated that the corresponding delay time is about 6h which appears to be in the right order of magnitude compared to the dynamics presented in [57]. However, the overall impact of the delay on model dynamics is limited. At least for the scenarios considered in the present paper, it is not critical for the quality of the agreement of model and data.

Furthermore, some adjustments were performed regarding chemotherapy modelling. Instead of an instantaneous cytotoxic effect of chemotherapy assumed in our former model version, we now assume a delayed effect to account for available data of the dynamics of bone marrow cell stages after chemotherapy applications in mice [58]. The delay parameter was assumed to be constant for all cell stages but specific for the applied drugs or drug combinations and was modelled by a cascade of first order transitions. This modelling is rather a phenomenological than a mechanistic approach since the delay is caused by many factors such as toxification of the applied drugs at different time scales, transient cell cycle arrests of cells and delayed apoptosis of cells due to irreversible damage [58].

Another improvement of our model is due to a semi-explicite modelling of lymphocyte toxicity. This was necessary in order to apply the model to a sufficiently large dataset of time courses of both, granulocytes and leukocytes as well. In our former model, we assumed proportionality of granulocytes and leukocytes during G-CSF application or chemotherapy which is only roughly correct [38] and further unpublished data of our chemotherapy studies). To avoid a complete cell kinetic model of lymphopoiesis requiring a large set of new and unknown model parameters, lymphocyte counts were modelled by a separate simple characteristic. We assumed no effect of G-CSF on lymphopoiesis but a toxic effect of chemotherapy modelled by an exponential depletion of lymphocytes according to a (delayed) chemotherapy toxicity function. This toxicity is again specific for the drugs or drug combinations applied. The resulting model was able to explain the time courses of leukocytes and granulocytes under G-CSF or chemotherapy adequately within the framework of one model.

The model is based on a relatively large set of parameters. Due to missing bone marrow data during chemotherapy and growth factor application, only a limited knowledge regarding the required cell-kinetic and toxicity parameters is available. Rough ranges for transition times and amplification rates in steady-state or under stimulation by G-CSF were obtained from the literature. But especially values under minimal stimulation, the sensitivity parameters of the Z-functions and the toxicity parameters are not available from literature data. Hence, many model parameters were determined indirectly by fitting the predictions of the model to available datasets. For this purpose, we collected a set of suitable data from the literature and clinical trials for which we have access to raw data. Densely measured time courses of G-CSF serum levels after application in combination with granulocyte or leukocyte counts after chemotherapy and different G-CSF schedules are especially useful. Data of patients were pooled to construct a model that fits to the median of patients. A unique parameter set was identified which is valid for all scenarios considered. No adjustments were performed in order to fit single scenarios. Not all datasets were used for model fitting enabling an opportunity for model validation.

Despite of the utilization of several datasets comprising different G-CSF dosing and timing schedules with and without chemotherapy, there remained a large uncertainty regarding parameter estimates, and consequently, the current parameter settings must be considered as preliminary. This is especially true for parameters with a low impact on our fitness function in the scenarios considered as demonstrated by our sensitivity analysis. Additionally, the toxicity parameters show some degree of dependence in the sense that a higher toxicity at one cell stage can to some degree be compensated by a lower toxicity at a subsequent cell stage and vice versa. Consequently, further datasets and validation scenarios are required to improve the confidence regarding our parameter settings.

The estimates of our pharmacokinetic parameters resulted in a good fit of all time series data of G-CSF serum concentration for both Filgrastim and Pegfilgrastim applications as well. The estimated values fit well to our biological understanding of the drugs. Due to the pegylation of the drug, it was expected that the unspecific renal clearance is significantly reduced for Pegfilgrastim, which is in agreement with our parameter estimates. We also estimated a reduced specific degradation of Pegfilgrastim which could be explained by a reduced receptor binding affinity or hydrophilic properties of pegylated molecules [26,27,76]. We also made the same observation for our pharmacokinetic models constructed in mice and rats [29,36]. Protein binding was estimated to be almost negligible for Filgrastim but important for Pegfilgrastim in agreement with our observations in mice [36]. Finally, the estimated distribution volumes are in rough agreement with findings of other authors [50,77].

The estimates of our cell-kinetic, pharmacodynamic and toxicity parameters also resulted in a good fit of the time courses of granulocytes and leukocytes after application of chemotherapy, G-CSF or combinations of it. Possible exceptions are cell counts measured shortly after the first application of Filgrastim, which seem to be underestimated in some scenarios. We conclude that the model works well on a day-wise scale but might be unable to explain short-term or transient effects of G-CSF applications e.g. on the scale of hours. Modelling such short-term effects would require a better database, since almost all available time courses of granulocytes and leukocytes were measured at most at a day-wise scale. However, in order to make predictions regarding the efficiency of different G-CSF schedules, we are also more interested in the long-term dynamics of granulocytes and leukocytes in the course of the therapy rather than short-term effects after single injections.

Estimates of the pharmacodynamic parameters of Filgrastim and Pegfilgrastim suggest that Filgrastim has a higher potency to stimulate the bone marrow. This is in agreement with our biological understanding that pegylation reduces the receptor binding affinity. An analogous observation was made for Pegfilgrastim and the novel drug Maxy-G34 which has even more pegylation sites than Pegfilgrastim [29].

Although modelling of chemotherapy was not the primary goal of the present paper, it was necessary to model at least a few conventional chemotherapy regimen to study the pharmacokinetic properties of the G-CSF derivatives under granulopenic conditions. Chemotherapy was modelled as a transient delayed toxic effect on all bone marrow cell stages. Corresponding toxicity parameters are specific for the bone marrow cell stages and the drugs and drug concentrations used. For the development of our model, we used the data of the most simple chemotherapy regimen CHOP. With our toxicity parameter estimates one obtains a good fit of all CHOP regimen with different G-CSF schedules.

Other conventional chemotherapies were modelled by assuming different toxicity parameters but the same cell kinetic model. Corresponding model simulations will be demonstrated in a separate paper of our group comprising about 20 different chemotherapy scenarios. Generally, the model can be applied to arbitrary conventional chemotherapy regimens for which data of leukocyte or granulocyte time courses are available for at least one G-CSF scheduling. Based on these data, sets of toxicity parameters of corresponding chemotherapies can be estimated. Using these parameters, it is possible to make clinically relevant predictions regarding the time course of G-CSF serum concentrations, bone marrow cell stages and mature cell counts in circulation under different G-CSF schedules, allowing to optimize G-CSF treatment. We will exploit the clinically relevant applications of our model in the near future.

## Conclusions

We established a human pharmacokinetic and -dynamic model of Filgrastim and Pegfilgrastim applications under cytotoxic chemotherapy. The model is able to explain a large number of clinical time series data of G-CSF serum concentrations, granulocytes and leukocytes of patients treated with G-CSF and with or without chemotherapy. A unique parameter set valid for all scenarios was established by fitting the predictions of the model to clinical data. The model was validated on a set of scenarios not used for parameter fitting. Differences between Filgrastim and Pegfilgrastim could be traced back to biologically plausible differences in parameter estimates. Effects of chemotherapy can be quantified by a set of toxicity parameters. Given these toxicity parameters, the model can be used to simulate the dynamics of G-CSF, bone marrow cell stages and circulating granulocytes or leukocytes of yet untested G-CSF schedules. The model is currently applied in the planning phase of clinical trials in order to optimize G-CSF treatment.

## Competing interests

The authors declare that they have no competing interests.

## Authors’ contributions

Model development: S.S., M.W., C.E., M.S. Parameter estimation and model simulations: S.S. Paper writing: M.S. All authors contributed to the discussion and the paper writing. All authors read and approved the final manuscript.

## Supplementary Material

Additional file 1 **Supplement Material.** Complete list of model equations, complete list of model parameters, additional model and data comparisons, sensitivity analysis [8,40,64,78-80]. Click here for file
