# Supplementary material for: Pharmacokinetic and -dynamic modelling of G-CSF derivatives in humans
Source: Theor Biol Med Model. 2012 Jul 30;9:32. doi: 10.1186/1742-4682-9-32 (PMC3507764; doi:10.1186/1742-4682-9-32)
Supplement: Additional file 1 — Supplement Material. Complete list of model equations, complete list of model parameters, additional model and data comparisons, sensitivity analysis [8,40,64,78-80]. [file 1742-4682-9-32-S1.pdf]

Pharmacokinetic and -dynamic modelling of G-CSF derivatives in humans

M. Scholz[1,2], S. Schirm[1], M. Wetzler[1], C. Engel[1,2], M. Loeffler[1,2]

[1]Institute for Medical Informatics, Statistics and Epidemiology, University of Leipzig, Haertelstrasse 16-18, 04107 Leipzig, Germany
[2] LIFE - Leipzig Research Center for Civilization Diseases, University of Leipzig, Philipp-Rosenthal-Strasse 27, 04103 Leipzig, Germany

Supplement material

A.1. Model Equations

Here we present all equations of the model except for the equations of the pharmacokinetic model which are explained in the main text. Initial conditions of all equations can be derived from steady-state conditions. Relative quantities are denoted by "*rel*" in the following and are obtained by dividing the compartment content by its steady-state value.

Modelling of Delays

Several delays are included into the model such as delayed action of G-CSF and chemotherapy. According to [1], these delays are modelled by a set of concatenated compartments with first order transitions

$$\text{(A.1)} \qquad \frac{d}{dt}C_X^1 = C_X - D_X C_X^1$$

$$\text{(A.2)} \qquad \frac{d}{dt}C_X^i = D_X\left(C_X^{i-1} - C_X^i\right) \quad i = 2, \ldots, N_X$$

where C_X is a quantity to be delayed, D_X is the delay parameter and N_X is the number of delay compartments. The delayed quantity is now defined by

$$\text{(A.3)} \qquad C_X^{del} = D_X C_X^{N_X}$$

Proliferative fraction

The proliferative fraction of the compartments S and CG is regulated by the complete granulopoietic system [1, 2]. Define

$$\text{(A.4)} \qquad x = \omega_G \ln C_G^{rel}(t) + \omega_S \cdot \begin{cases} \ln C_S^{rel}(t) & \text{for } C_S^{rel} \leq 1 \\ C_S^{rel}(t) - 1 & \text{for } C_S^{rel} > 1 \end{cases}$$

$$\text{(A.5)} \qquad y = -\frac{1}{2\ln 2}\left(\ln\left(\frac{a_X^{int} - a_X^{max}}{a_X^{min} - a_X^{int}}\right) - \ln\left(\frac{a_X^{nor} - a_X^{max}}{a_X^{min} - a_X^{nor}}\right)\right) x + \frac{1}{2}\ln\left(\frac{a_X^{nor} - a_X^{max}}{a_X^{min} - a_X^{nor}}\right)$$

then the proliferative fraction is given by

$$\text{(A.6)} \qquad a_X = \begin{cases} \frac{a_X^{max} e^{-y} + a_X^{min} e^{y}}{e^{-y} + e^{y}} & \text{for } a_X^{min} < a_X^{int} < a_X^{nor} < a_X^{max} \\ a_X^{nor} & \text{for } a_X^{min} = a_X^{int} = a_X^{nor} = a_X^{max} \end{cases} .$$

where X is either S or CG. This is a monotonically increasing function ranging between a_X^{min} and a_X^{max}. The values a_X^{nor} and a_X^{int} correspond to normal and intensified granulopoiesis respectively. The parameters ω_S and ω_G are weighting factors representing the strengthes of influence of stem cells and granulopoietic cells on the proliferative fraction.

Compartment S

The self-renewal probability of stem cells p is regulated by a competition of granulopoietic cells and the stem cell content [1, 2]. Define

$$\text{(A.7)} \qquad \vartheta_S(t) = \begin{cases} \frac{2}{C_S^{rel}(t)^{0.6}} & \text{for } C_S^{rel} \leq 1 \\ 2 & \text{for } C_S^{rel} > 1 \end{cases}$$

then the self-renewal probability is given by

$$\text{(A.8)} \qquad p = p_\delta \tanh\left(-\vartheta_S(t)\left(C_S^{rel}(t) - 1\right) - \vartheta_G\left(C_G^{rel}(t) - 1\right)\right) + 0.5$$

The weighting factors ϑ_S and ϑ_G represent the strengths of influence of stem cells and granulopoietic cells on the self-renewal probability. Due to equation (A.7), self-renewal of stem cells is given priority if the number of stem cells becomes small. The parameter p_δ represents the range of possible adaptations of the self-renewal probability.
Equations of stem cell dynamics are given by

$$\text{(A.9)} \qquad \frac{d}{dt}C_S = (2p - 1)\, C_S \frac{a_S}{\tau_S} - k_S \Psi_{CX} C_S$$

$$\text{(A.10)} \qquad C_S^{out} = 2\,(1 - p)\, C_S \frac{a_S}{\tau_S}$$

where Ψ_{CX} is the characteristic chemotherapy function. This function is a delayed step-function with a step-length of one day for each chemotherapy appliction. The

parameters k_X are the toxicity parameters of the corresponding cell stages X. The parameters k_X are constant during the course of the therapy except for the first chemotherapy application for which a greater toxicity is assumed by multiplying k_X with the first-cycle effect $f_{fc} \geq 1$ (see [1] for further details).

Compartment CG

$$
\begin{aligned}
&\text{(A.11)} & A_{CG} &= Z_{A_{CG}}\left(C_{G-CSF}^{cent\_rel\_del}\right)\\
&\text{(A.12)} & T_{CG} &= Z_{T_{CG}}\left(C_{G-CSF}^{cent\_rel\_del}\right)\\
&\text{(A.13)} & \frac{d}{dt}C_{CG} &= C_S^{out}A_{CG}^{in} - C_{CG}\frac{a_{CG}}{T_{CG}} - k_{CG}\Psi_{CX}C_{CG}\\
&\text{(A.14)} & C_{CG}^{out} &= C_{CG}A_{CG}^{out}\frac{a_{CG}}{T_{CG}}
\end{aligned}
$$

Both, A_{CG} and T_{CG} are regulated by the delayed concentration of G-CSF.

Compartment PGB

$$
\begin{aligned}
&\text{(A.15)} & A_{PGB} &= Z_{A_{PGB}}\left(C_{G-CSF}^{cent\_rel\_del}\right)\\
&\text{(A.16)} & T_{PGB} &= Z_{T_{PGB}}\left(C_{G-CSF}^{cent\_rel\_del}\right)\\
&\text{(A.17)} & \frac{d}{dt}C_{PGB} &= C_{CG}^{out}A_{PGB}^{in} - C_{PGB}\frac{a_{PGB}}{T_{PGB}} - k_{PGB}\Psi_{CX}C_{PGB}\\
&\text{(A.18)} & C_{PGB}^{out} &= C_{PGB}A_{PGB}^{out}\frac{a_{PGB}}{T_{PGB}}
\end{aligned}
$$

Compartment MGB

This compartment is divided into three compartments denoted as $G4$, $G5$ and $G6$. The compartments are again divided into N_X subcompartments to model the maturation process by a delay. In these subcompartments, the effect of postmitotic apoptosis is implemented [1, 3] by introducing a postmitotic amplification detoned again as A. It

holds that $A \leq 1$ for all subcompartments.

$$
\begin{aligned}
&\text{(A.19)} \quad & C_{MGB} &= C_{G4} + C_{G5} + C_{G6} \\
&\text{(A.20)} \quad & C_{MGB}^{out} &= C_{C6}^{out} \\
&\text{(A.21)} \quad & A_{G4} &= Z_{A_{G4}}\left(C_{G-CSF}^{cent\_rel\_del}\right) \\
&\text{(A.22)} \quad & T_{G4} &= Z_{T_{G4}}\left(C_{G-CSF}^{cent\_rel\_del}\right) \\
&\text{(A.23)} \quad & C_{G4} &= \sum_{i=1}^{N_{G4}} C_{G4\_i} \\
&\text{(A.24)} \quad & \frac{d}{dt}C_{G4\_1} &= C_{PGB}^{out} - C_{G4\_1}\frac{N_{G4}}{T_{G4}} - k_{MGB}\Psi_{CX}C_{G4\_1} \\
&\text{(A.25)} \quad & \frac{d}{dt}C_{G4\_i} &= C_{G4\_(i-1)}^{out} - C_{G4\_i}\frac{N_{G4}}{T_{G4}} - k_{MGB}\Psi_{CX}C_{G4\_i}, \quad i = 2,\ldots,N_{G4} \\
&\text{(A.26)} \quad & C_{G4\_i}^{out} &= A_{G4\_i}C_{G4\_i}\frac{N_{G4}}{T_{G4}}, \quad i = 1,\ldots,N_{G4} \\
&\text{(A.27)} \quad & C_{G4}^{out} &= C_{G4\_N_{G4}}^{out}
\end{aligned}
$$

The total postmitotic amplification A_{G4} is equally distributed over all subcompartments in which there is postmitotic amplification, e.g. if there is postmitotic amplification in all subcompartments, it holds that $A_{G4\_i} = {A_{G4}}^{1/N_{G4}}$.

For $G5$ and $G6$ the equations are completely analogous to (A.21)-(A.27) if one replaces PGB by $G4$ and $G5$ respectively. In the present form of our model, postmitotic amplification is restricted to $G6$.

Compartment GRA

$$
\begin{aligned}
&\text{(A.28)} \quad & \frac{d}{dt}C_{GRA} &= C_{MGB}^{out} - C_{GRA}\frac{1}{T_{GRA}} \\
&\text{(A.29)} \quad & T_{GRA} &= T_{GRA}^{nor}\left(1 + T_{GRA}^{Pred}\Psi_{Pred}\right)
\end{aligned}
$$

where Ψ_{Pred} is the characteristic function of Prednisone applications modelled as a step-function which is equal to one for the duration of one day after Prednisone application.

Granulopoietic cells

$$
\text{(A.30)} \quad C_G = C_{CG} + C_{PGB} + C_{MGB}
$$

A.2. Model Parameters

In this section we present a complete list of our model parameters, separated for the relevant compartments.

Compartment S

parameter/ quantity	meaning	value	source
S^{nor}	normal value of stem cells	1	set
τ_S	duration of cell cycle	8	WL*
p_δ	self-renewal probability	0.1	WL
a_S^{min}	proliferative fraction under minimal stimulation	0.01	WL
a_S^{nor}	proliferative fraction under normal stimulation	0.15	WL
a_S^{int}	proliferative fraction under intensified stimulation	0.45	WL
a_S^{max}	proliferative fraction under maximal stimulation	1	WL
ω_G	weighting parameter G for regulation of a	0.4	WL
ω_S	weighting parameter S for regulation of a	1	WL
ϑ_G	weighting parameter G for regulation of p	-10	WL

*[2]

Compartment CG

parameter/ quantity	meaning	value	source
a_{CG}^{min}	proliferative fraction under minimal stimulation	0.1205	fitted
a_{CG}^{nor}	proliferative fraction under normal stimulation	0.1252	fitted
a_{CG}^{int}	proliferative fraction under intensified stimulation	0.8340	fitted
a_{CG}^{max}	proliferative fraction under maximal stimulation	1	WL*

*[2]

Compartment MGB

parameter/ quantity	meaning	value	source
N_{G4}	number of subcompartments in $G4$	5	set
N_{G5}	number of subcompartments in $G5$	5	set
N_{G6}	number of subcompartments in $G6$	5	set

Compartment GRA

parameter/ quantity	meaning	value	source
T_{GRA}^{nor}	transition time of granulocytes	5.576	fitted, [4]
T_{GRA}^{pred}	prolongation of T_{GRA}^{nor} under Prednisone	0.466	fitted, [5]

Compartment endogenous $G - CSF$

parameter/ quantity	meaning	value	source
$P_{G-CSF}^{endo\_max}$	maximal G-CSF production	257	fitted
$P_{G-CSF}^{endo\_nor}$	normal G-CSF production	1	set
$P_{G-CSF}^{endo\_min}$	minimal G-CSF production	0.318	fitted
$P_{G-CSF}^{endo\_b}$	sensitivity parameter of G-CSF production	0.022	fitted
ω_{GRA}	influence of GRA on G-CSF production	1	set, [1]
ω_{G6}	influence of $G6$ on G-CSF production	0.2	set, [1]

Pharmacokinetic parameters of Filgrastim/endogenous G-CSF

parameter/ quantity	meaning	value	source
k_{sc}^F	subcutaneous absorption	0.161	fitted
k_m^F	Michaelis-Menten constant of subcutaneous elimination	34.7	fitted
v_{max}^F	Maximum of subcutaneous elimination	67.3	fitted
k_u^F	unspecific elimination	0.441	fitted
k_{cp}^F	transition central to peripheral	0.000	fitted
k_{pc}^F	transition peripheral to central	-	not determinable
V_D^F	distribution volume	1.156	fitted
$v_{max}^{GRA\_F}$	Maximum of specific elimination	4.77	fitted
$k_m^{GRA\_F}$	Michaelis-Menten constant of specific elimination	22.4	fitted

Pharmacodynamic parameters of Filgrastim/endogenous G-CSF

parameter/ quantity	meaning	value	source
Compartment CG			
$A_{CG_F}^{min}$	amplification in CG under minimal stimulation	0.910	fitted
$A_{CG_F}^{nor}$	amplification in CG under normal stimulation	105	fitted
$A_{CG_F}^{max}$	amplification in CG under maximal stimulation	206	fitted
$A_{CG_F}^{b}$	sensitivity of amplification in CG	0.024	fitted
$T_{CG_F}^{min}$	transition time in CG under minimal stimulation	47.3	fitted
$T_{CG_F}^{nor}$	transition time in CG under normal stimulation	78.2	fitted
$T_{CG_F}^{max}$	transition time in CG under maximal stimulation	286	fitted
$T_{CG_F}^{b}$	sensitivity of transition time in CG	0.590	fitted
Compartment PGB			
$A_{PGB_F}^{min}$	amplification in PGB under minimal stimulation	1.31	fitted
$A_{PGB_F}^{nor}$	amplification in PGB under normal stimulation	61.2	fitted
$A_{PGB_F}^{max}$	amplification in PGB under maximal stimulation	815	fitted

$A^b_{PGB_F}$	sensitivity of amplification in PGB	0.721	fitted
$T^{min}_{PGB_F}$	transition time in PGB under minimal stimulation	4.64	fitted
$T^{nor}_{PGB_F}$	transition time in PGB under normal stimulation	40.9	fitted
$T^{max}_{PGB_F}$	transition time in PGB under maximal stimulation	217	fitted
$T^b_{PGB_F}$	sensitivity of transition time in PGB	0.104	fitted
Compartment MGB			
$A^{nor}_{G4_F}$	postmitotic amplification in $G4$	1	set
$T^{min}_{G4_F}$	transition time in $G4$ under minimal stimulation	119	fitted
$T^{nor}_{G4_F}$	transition time in $G4$ under normal stimulation	11.4	fitted
$T^{max}_{G4_F}$	transition time in $G4$ under maximal stimulation	3.93	fitted
$T^b_{G4_F}$	sensitivity of transition time in $G4$	0.366	fitted
$A^{nor}_{G5_F}$	postmitotic amplification in $G5$	1	set
$T^{min}_{G5_F}$	transition time in $G5$ under minimal stimulation	48.3	fitted
$T^{nor}_{G5_F}$	transition time in $G5$ under normal stimulation	37.0	fitted
$T^{max}_{G5_F}$	transition time in $G5$ under maximal stimulation	4.64	fitted
$T^b_{G5_F}$	sensitivity of transition time in $G5$	0.459	fitted
$A^{min}_{G6_F}$	postmitotic amplification in $G6$ under minimal stimulation	0.201	fitted
$A^{nor}_{G6_F}$	postmitotic amplification in $G6$ under normal stimulation	0.249	fitted
$A^{max}_{G6_F}$	postmitotic amplification in $G6$ under maximal stimulation	0.850	fitted
$A^b_{G6_F}$	sensitivity of postmitotic amplification in $G6$	0.503	fitted
$T^{min}_{G6_F}$	transition time in $G6$ under minimal stimulation	141	fitted
$T^{nor}_{G6_F}$	transition time in $G6$ under normal stimulation	82.0	fitted
$T^{max}_{G6_F}$	transition time in $G6$ under maximal stimulation	41.4	fitted
$T^b_{G6_F}$	sensitivity of transition time in $G6$	0.526	fitted
Delay parameters			
D^F_{G-CSF}	Delay of Filgrastim action	1.08	fitted
N^F_{G-CSF}	number of delay compartments	4	set

Pharmacokinetic parameters of Pegfilgrastim

parameter/ quantity	meaning	value	source
k^P_{sc}	subcutaneous absorption	0.107	fitted
k^P_m	Michaelis-Menten constant of subcutaneous elimination	5.5	fitted
v^P_{max}	Maximum of subcutaneous elimination	16.5	fitted
k^P_u	unspecific elimination	0.087	fitted
k^P_{cp}	transition central to peripheral	0.075	fitted
k^P_{pc}	transition peripheral to central	0.548	fitted
V^P_D	distribution volume	4.091	fitted
$v^{GRA\_P}_{max}$	Maximum of specific elimination	5.16	fitted
$k^{GRA\_P}_m$	Michaelis-Menten constant of specific elimination	30.8	fitted

Pharmacodynamic parameters of Pegfilgrastim

parameter/ quantity	meaning	value	source
	Compartment CG		
$A_{CG_P}^{min}$	amplification in CG under minimal stimulation	4.21	fitted
$A_{CG_P}^{nor}$	amplification in CG under normal stimulation	357	fitted
$A_{CG_P}^{max}$	amplification in CG under maximal stimulation	669	fitted
$A_{CG_P}^{b}$	sensitivity of amplification in CG	0.103	fitted
$T_{CG_P}^{min}$	transition time in CG under minimal stimulation	0.848	fitted
$T_{CG_P}^{nor}$	transition time in CG under normal stimulation	1.69	fitted
$T_{CG_P}^{max}$	transition time in CG under maximal stimulation	57.6	fitted
$T_{CG_P}^{b}$	sensitivity of transition time in CG	0.040	fitted
	Compartment PGB		
$A_{PGB_P}^{min}$	amplification in PGB under minimal stimulation	0.067	fitted
$A_{PGB_P}^{nor}$	amplification in PGB under normal stimulation	24.5	fitted
$A_{PGB_P}^{max}$	amplification in PGB under maximal stimulation	47.0	fitted
$A_{PGB_P}^{b}$	sensitivity of amplification in PGB	0.327	fitted
$T_{PGB_P}^{min}$	transition time in PGB under minimal stimulation	0.756	fitted
$T_{PGB_P}^{nor}$	transition time in PGB under normal stimulation	24.7	fitted
$T_{PGB_P}^{max}$	transition time in PGB under maximal stimulation	24.7	fitted
$T_{PGB_P}^{b}$	sensitivity of transition time in PGB	0.118	fitted
	Compartment MGB		
$A_{G4_P}^{nor}$	postmitotic amplification in $G4$	1	set
$T_{G4_P}^{min}$	transition time in $G4$ under minimal stimulation	153	fitted
$T_{G4_P}^{nor}$	transition time in $G4$ under normal stimulation	14.2	fitted
$T_{G4_P}^{max}$	transition time in $G4$ under maximal stimulation	4.17	fitted
$T_{G4_P}^{b}$	sensitivity of transition time in $G4$	0.225	fitted
$A_{G5_P}^{nor}$	postmitotic amplification in $G5$	1	set
$T_{G5_P}^{min}$	transition time in $G5$ under minimal stimulation	153	fitted
$T_{G5_P}^{nor}$	transition time in $G5$ under normal stimulation	14.2	fitted
$T_{G5_P}^{max}$	transition time in $G5$ under maximal stimulation	4.17	fitted
$T_{G5_P}^{b}$	sensitivity of transition time in $G5$	0.225	fitted
$A_{G6_P}^{min}$	postmitotic amplification in $G6$ under minimal stimulation	0.139	fitted
$A_{G6_P}^{nor}$	postmitotic amplification in $G6$ under normal stimulation	0.368	fitted
$A_{G6_P}^{max}$	postmitotic amplification in $G6$ under maximal stimulation	1	set
$A_{G6_P}^{b}$	sensitivity of postmitotic amplification in $G6$	0.321	fitted
$T_{G6_P}^{min}$	transition time in $G6$ under minimal stimulation	153	fitted
$T_{G6_P}^{nor}$	transition time in $G6$ under normal stimulation	14.2	fitted
$T_{G6_P}^{max}$	transition time in $G6$ under maximal stimulation	4.17	fitted
$T_{G6_P}^{b}$	sensitivity of transition time in $G6$	0.225	fitted

Delay parameters and and competiton of receptor binding			
D^P_{G-CSF}	Delay of Pegfilgrastim action	0.984	fitted
N^P_{G-CSF}	number of delay compartments	4	set
ω_P^{min}	minimum of weighting function	0	set
ω_P^{nor}	value of weighting function for $1\mu g$ Pegfilgrastim	0.499	fitted
ω_P^{max}	maximum of weighting function	1	set
ω_P^{b}	sensitivity parameter of weighting function	0.068	fitted

Chemotherapy parameters for CHOP regimen

parameter/ quantity	meaning	value	source
f_{fc}	first cycle effect	1.09	fitted
D_{CX}	delay of toxicity	0.0486	fitted
k_S	toxicity in S compartment	0.212	fitted
k_{CG}	toxicity in CG compartment	0.464	fitted
k_{PGB}	toxicity in PGB compartment	0.168	fitted
k_{MGB}	toxicity in MGB compartment	0.000148	fitted
D_{CX_LYM}	delay of lymphocyte toxicity	0.0223	fitted
k_{LYM}	lymphocyte toxicity	16.4	fitted

A.3. Additional Figures

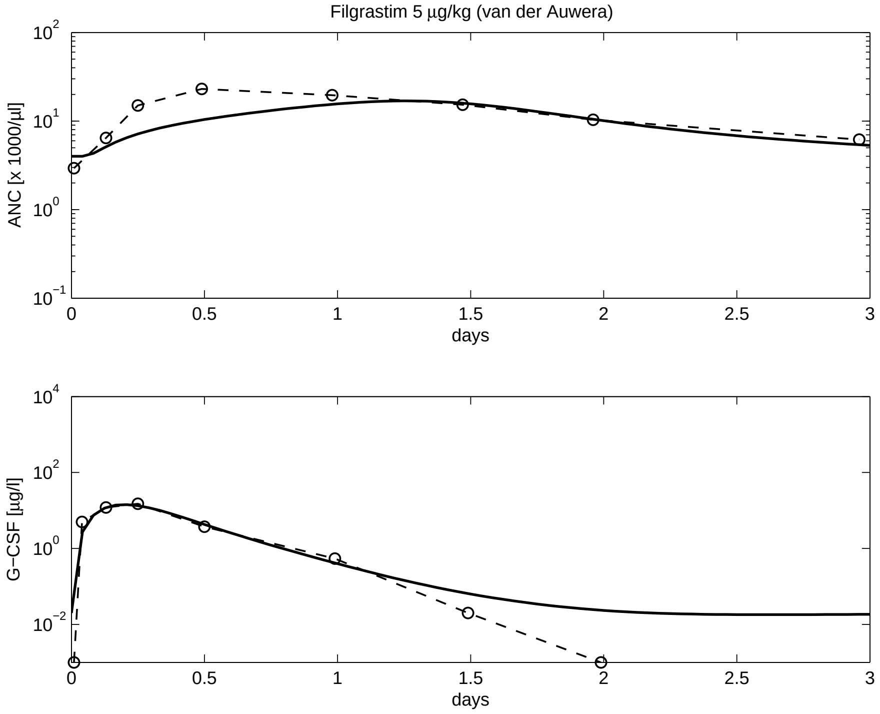

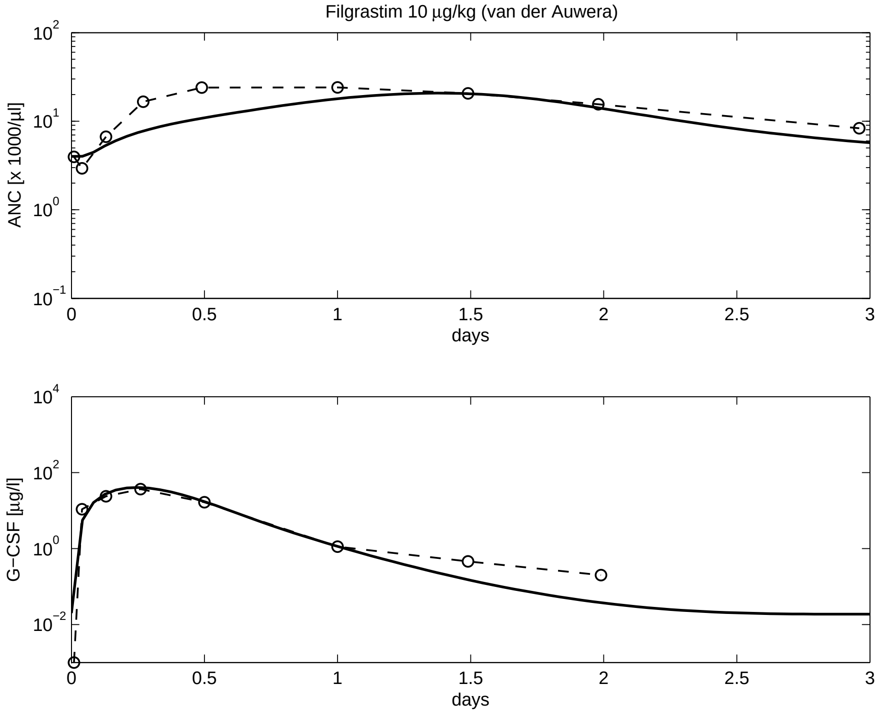

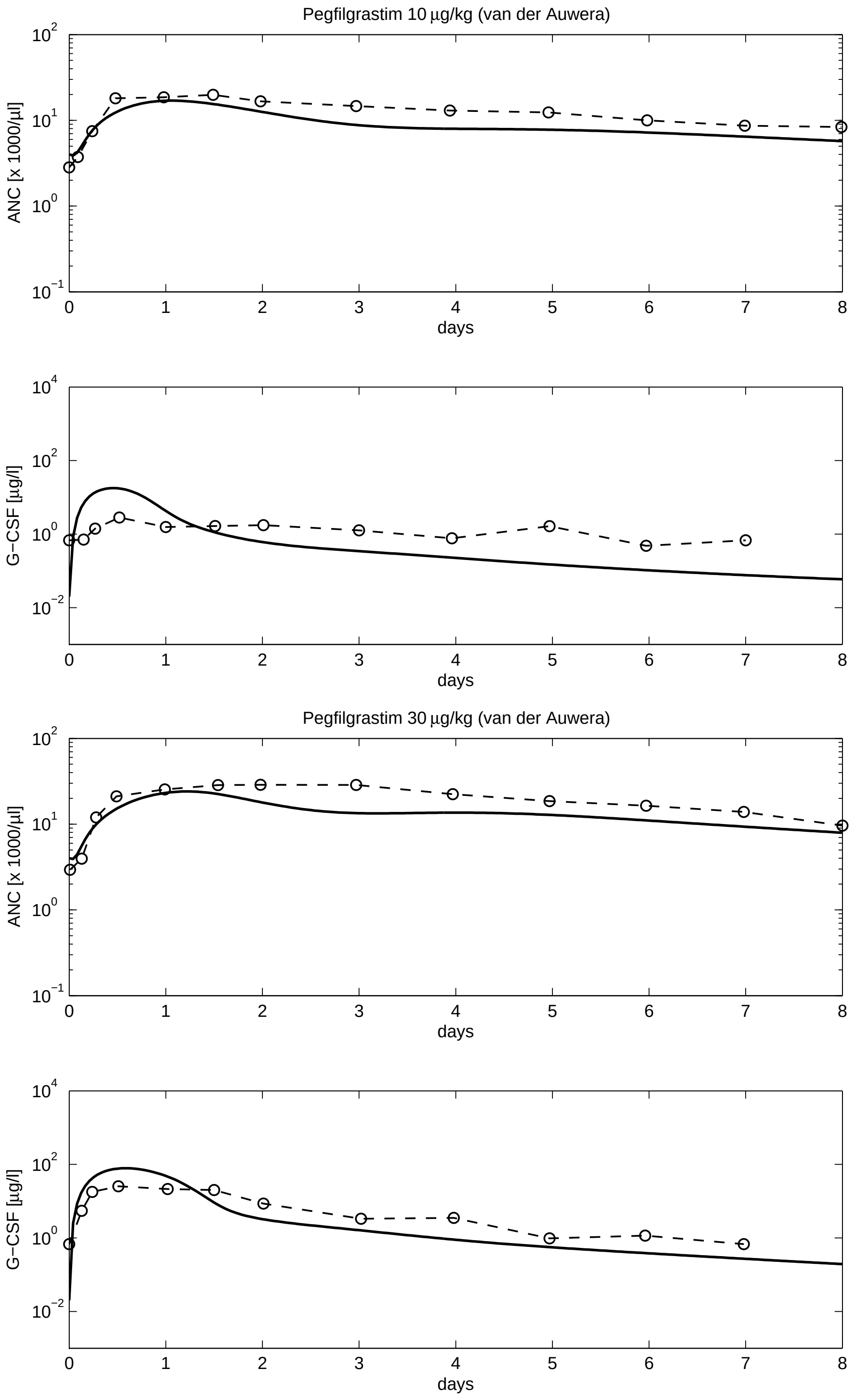
Pegfilgrastim 10 µg/kg (van der Auwera)
ANC [x 1000/µl]
G−CSF [µg/l]
days
Pegfilgrastim 30 µg/kg (van der Auwera)
ANC [x 1000/µl]
G−CSF [µg/l]
days

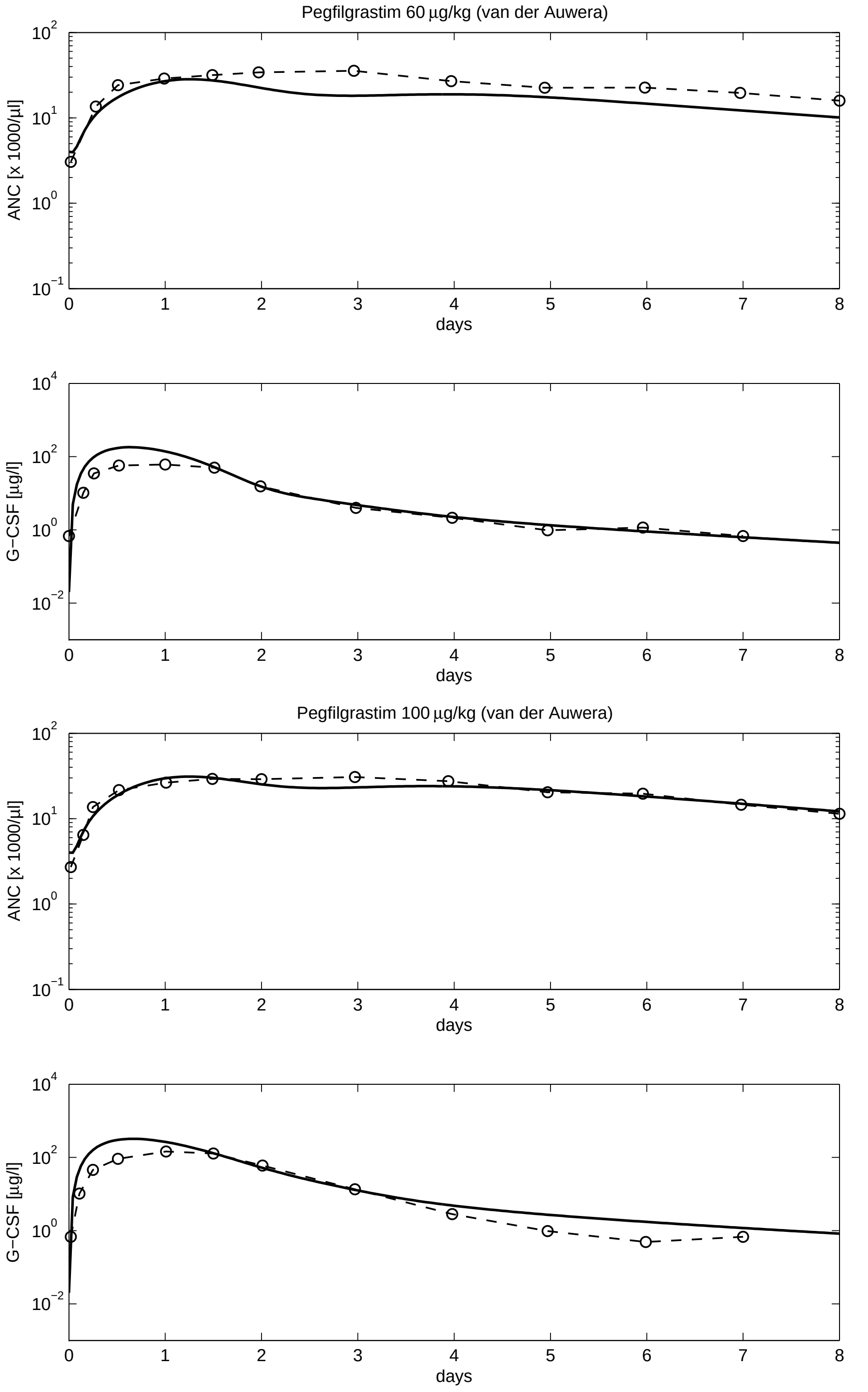
Pegfilgrastim 60 µg/kg (van der Auwera)
ANC [x 1000/µl]
10^2
10^1
10^0
10^{-1}
0 1 2 3 4 5 6 7 8
days
G−CSF [µg/l]
10^4
10^2
10^0
10^{-2}
0 1 2 3 4 5 6 7 8
days
Pegfilgrastim 100 µg/kg (van der Auwera)
ANC [x 1000/µl]
10^2
10^1
10^0
10^{-1}
0 1 2 3 4 5 6 7 8
days
G−CSF [µg/l]
10^4
10^2
10^0
10^{-2}
0 1 2 3 4 5 6 7 8
days

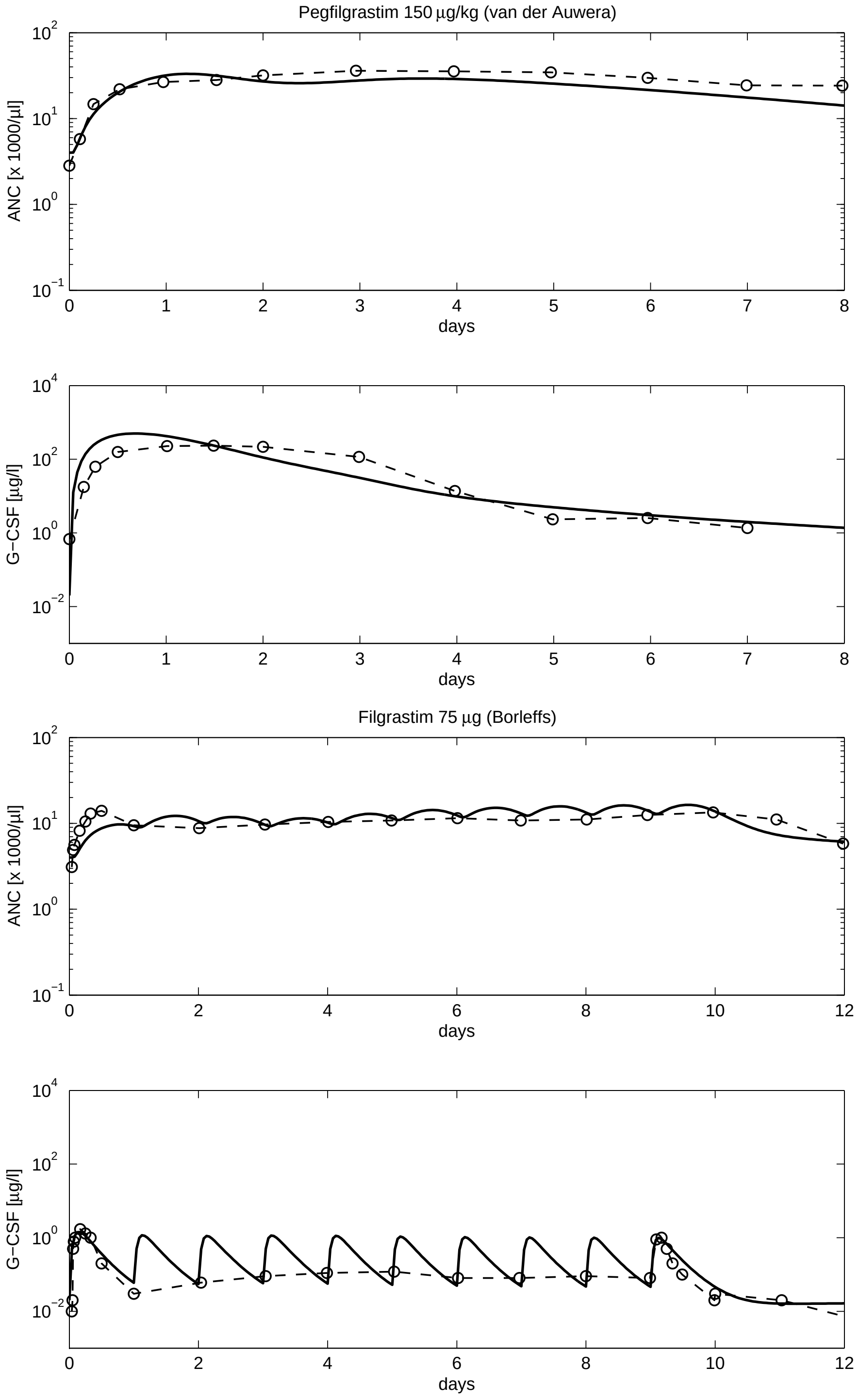

Pegfilgrastim 150 µg/kg (van der Auwera)
ANC [x 1000/µl]
G−CSF [µg/l]
days
Filgrastim 75 µg (Borleffs)
ANC [x 1000/µl]
G−CSF [µg/l]
days

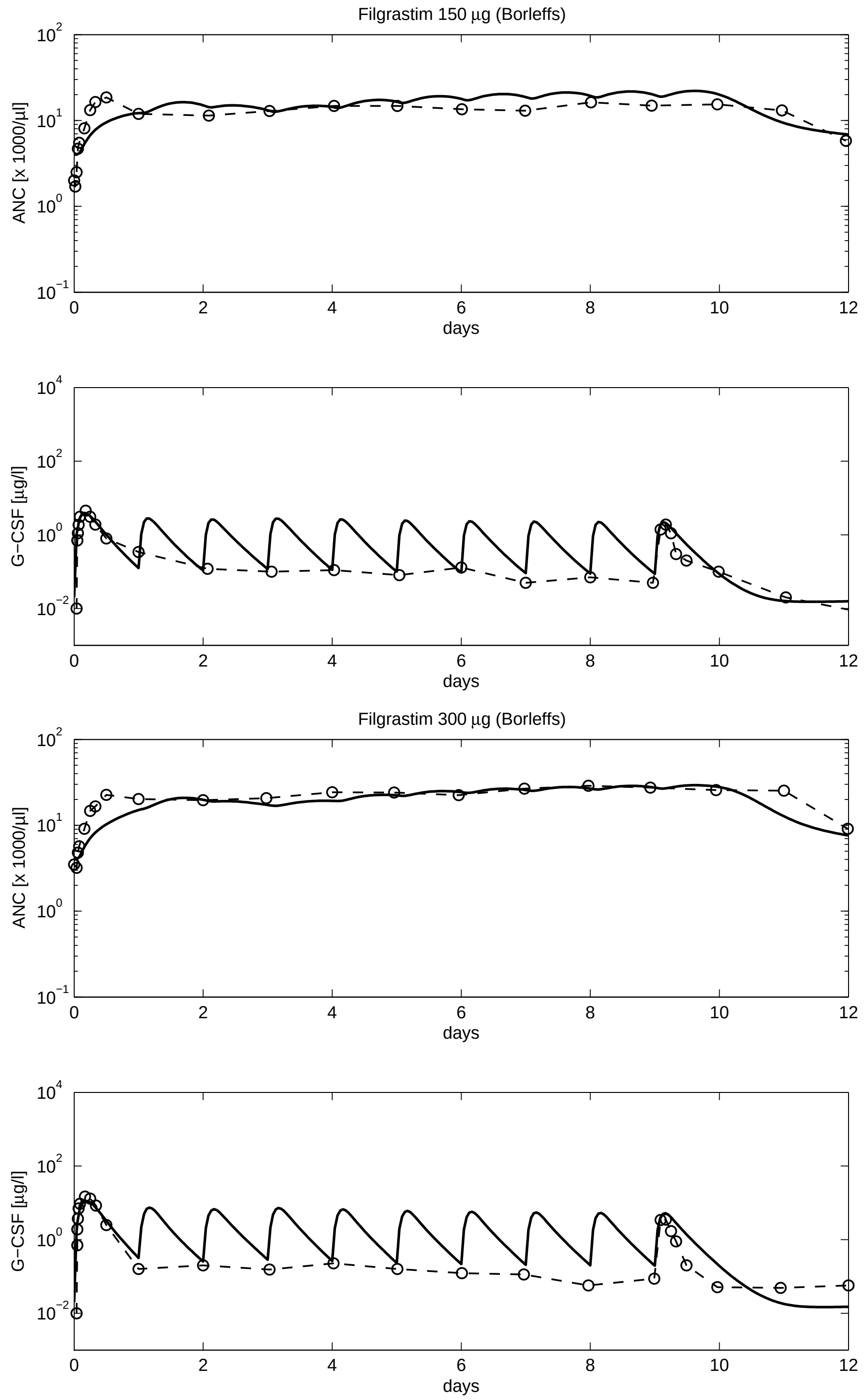
Filgrastim 150 µg (Borleffs)
ANC [x 1000/µl]
G−CSF [µg/l]
days
Filgrastim 300 µg (Borleffs)
ANC [x 1000/µl]
G−CSF [µg/l]
days

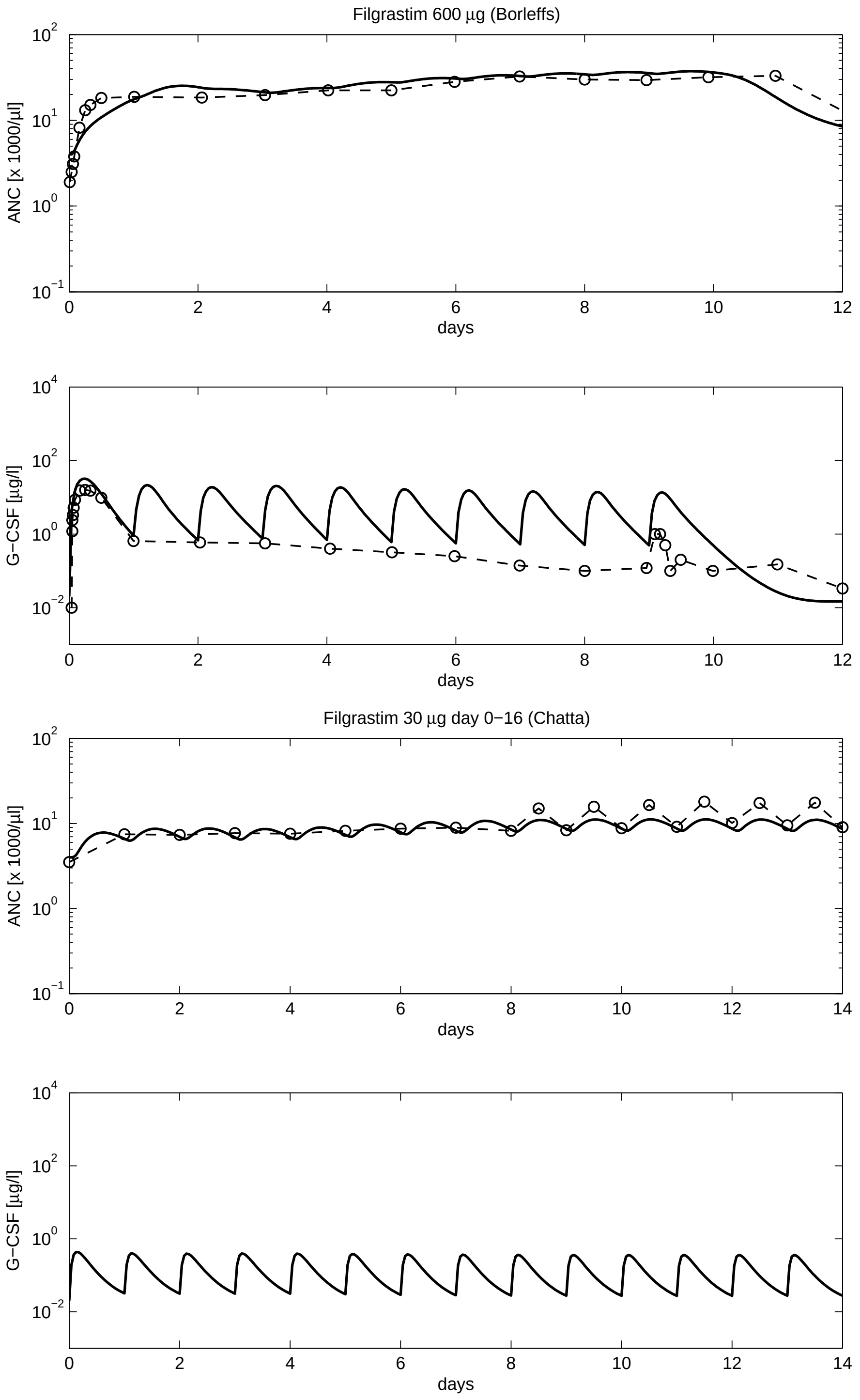
Filgrastim 600 µg (Borleffs)
ANC [x 1000/µl]
G−CSF [µg/l]
days
Filgrastim 30 µg day 0−16 (Chatta)
ANC [x 1000/µl]
G−CSF [µg/l]
days

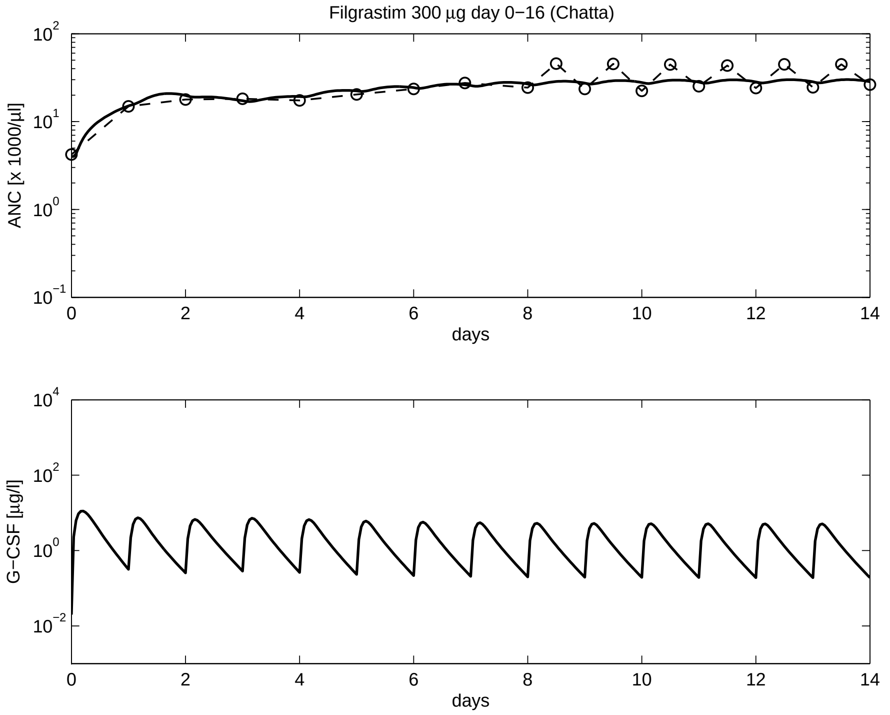
Filgrastim 300 µg day 0−16 (Chatta)
ANC [x 1000/µl]
days
G−CSF [µg/l]
days

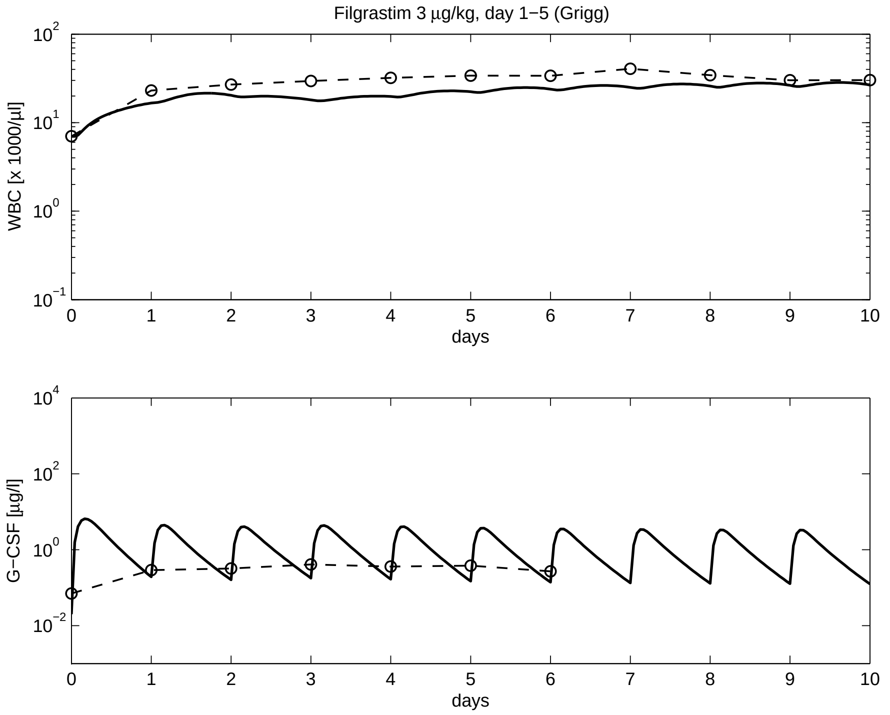
Filgrastim 3 µg/kg, day 1−5 (Grigg)
WBC [x 1000/µl]
days
G−CSF [µg/l]
days

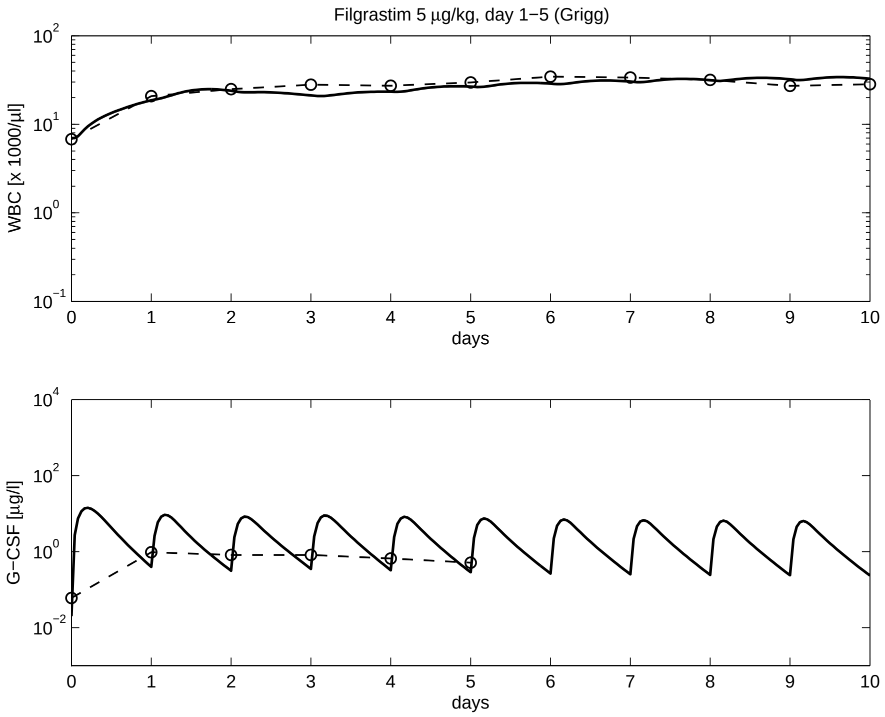
Filgrastim 5 µg/kg, day 1−5 (Grigg)
WBC [x 1000/µl]
days
G−CSF [µg/l]
days

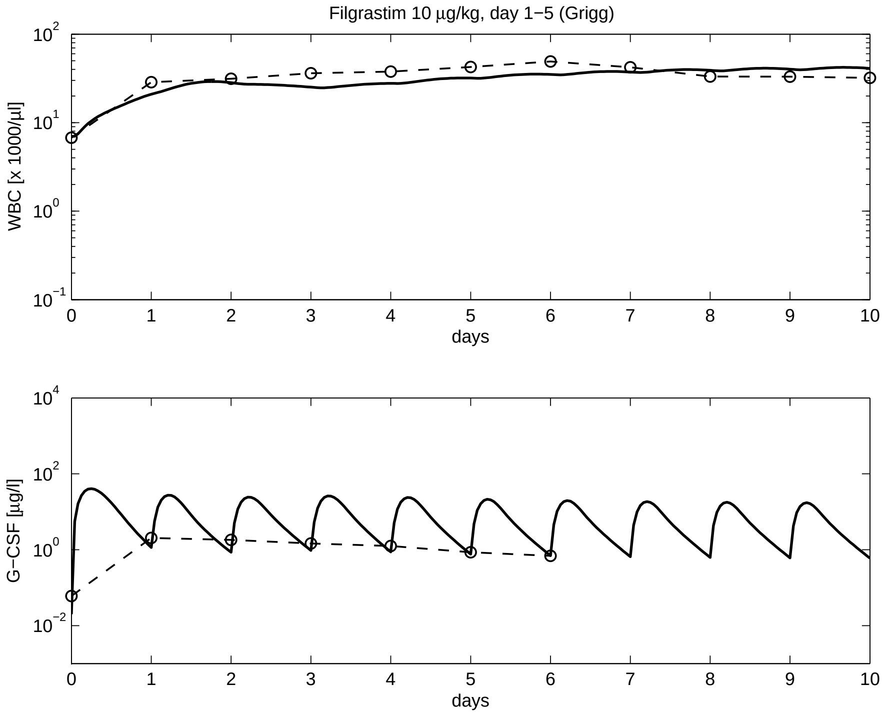
Filgrastim 10 µg/kg, day 1−5 (Grigg)
WBC [x 1000/µl]
days
G−CSF [µg/l]
days

Filgrastim 5 µg/kg, Carboplatin, Paclitaxel (Johnston)

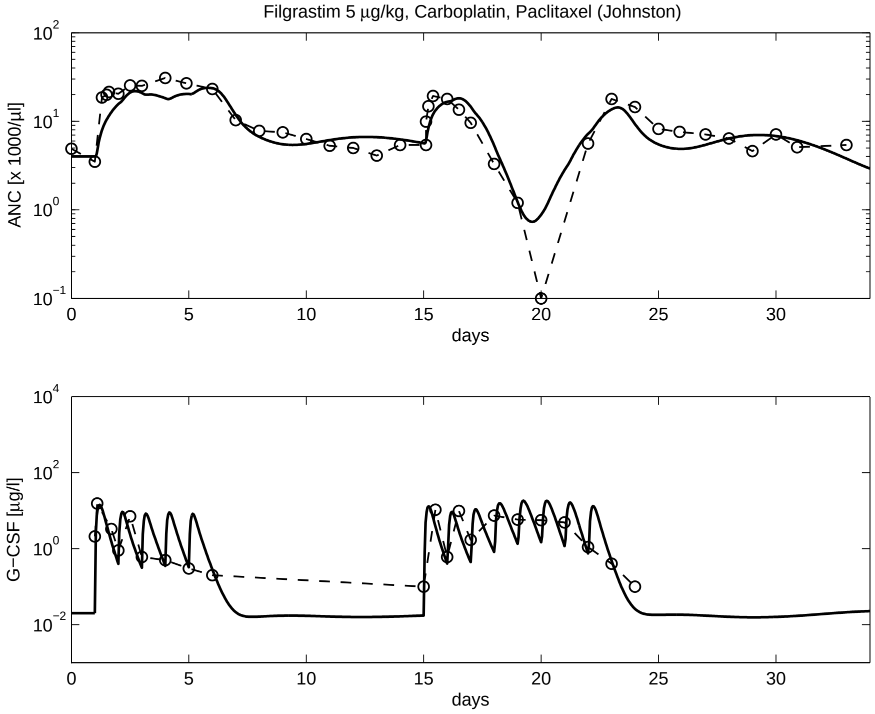

Pegfilgrastim 30 µg/kg, Carboplatin, Paclitaxel (Johnston)

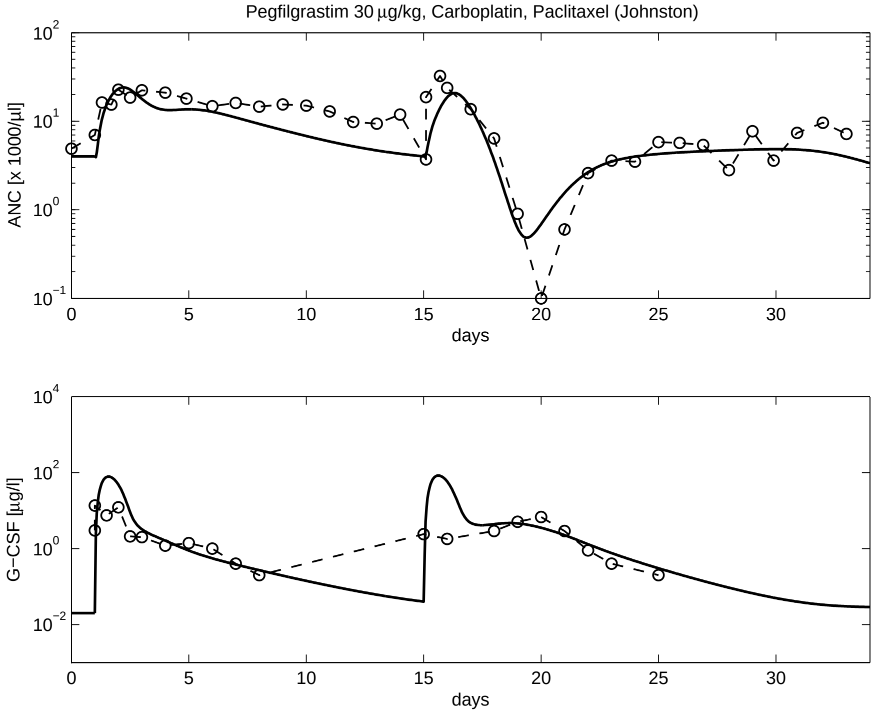

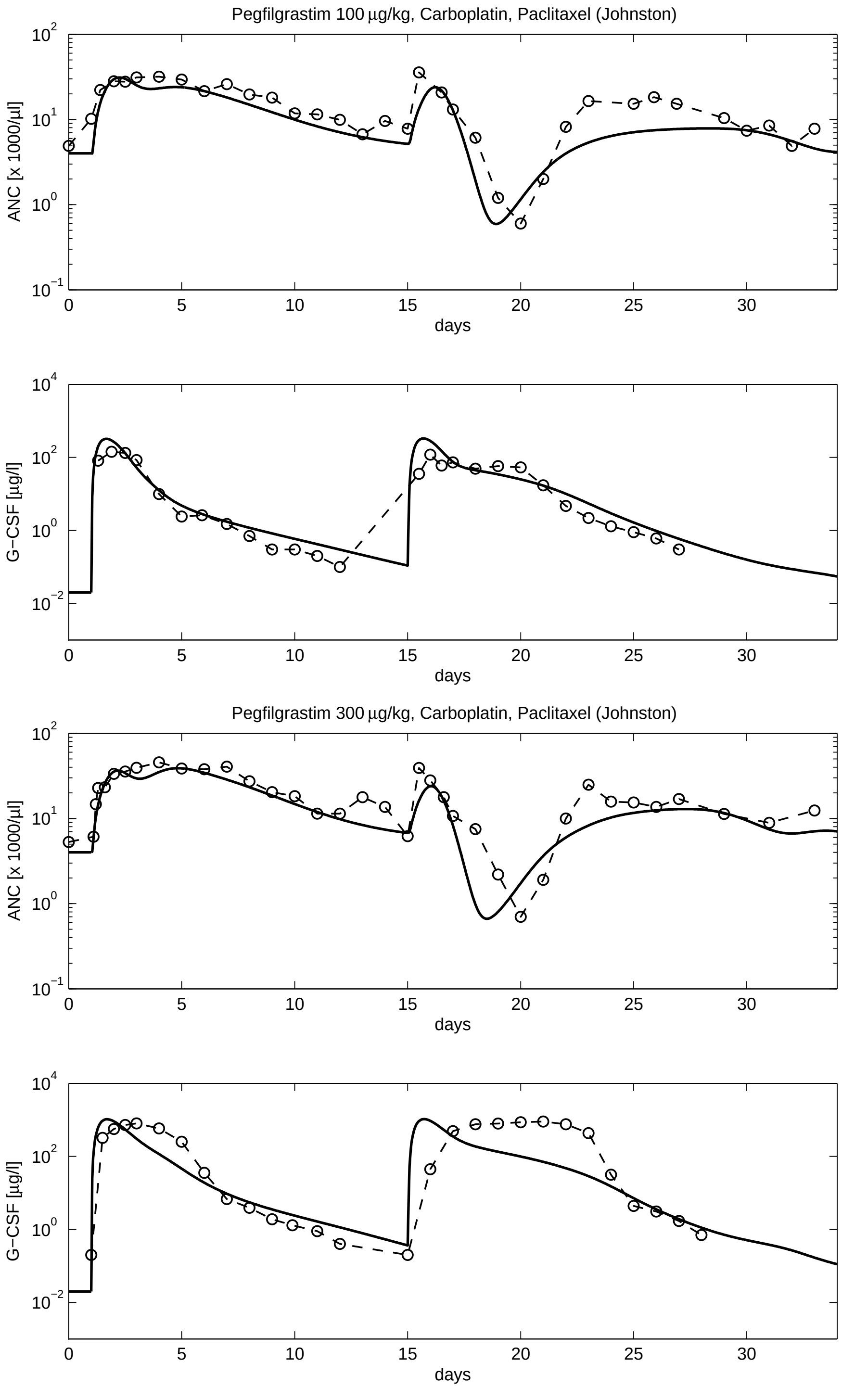
Pegfilgrastim 100 µg/kg, Carboplatin, Paclitaxel (Johnston)
ANC [x 1000/µl]
G−CSF [µg/l]
days
Pegfilgrastim 300 µg/kg, Carboplatin, Paclitaxel (Johnston)
ANC [x 1000/µl]
G−CSF [µg/l]
days

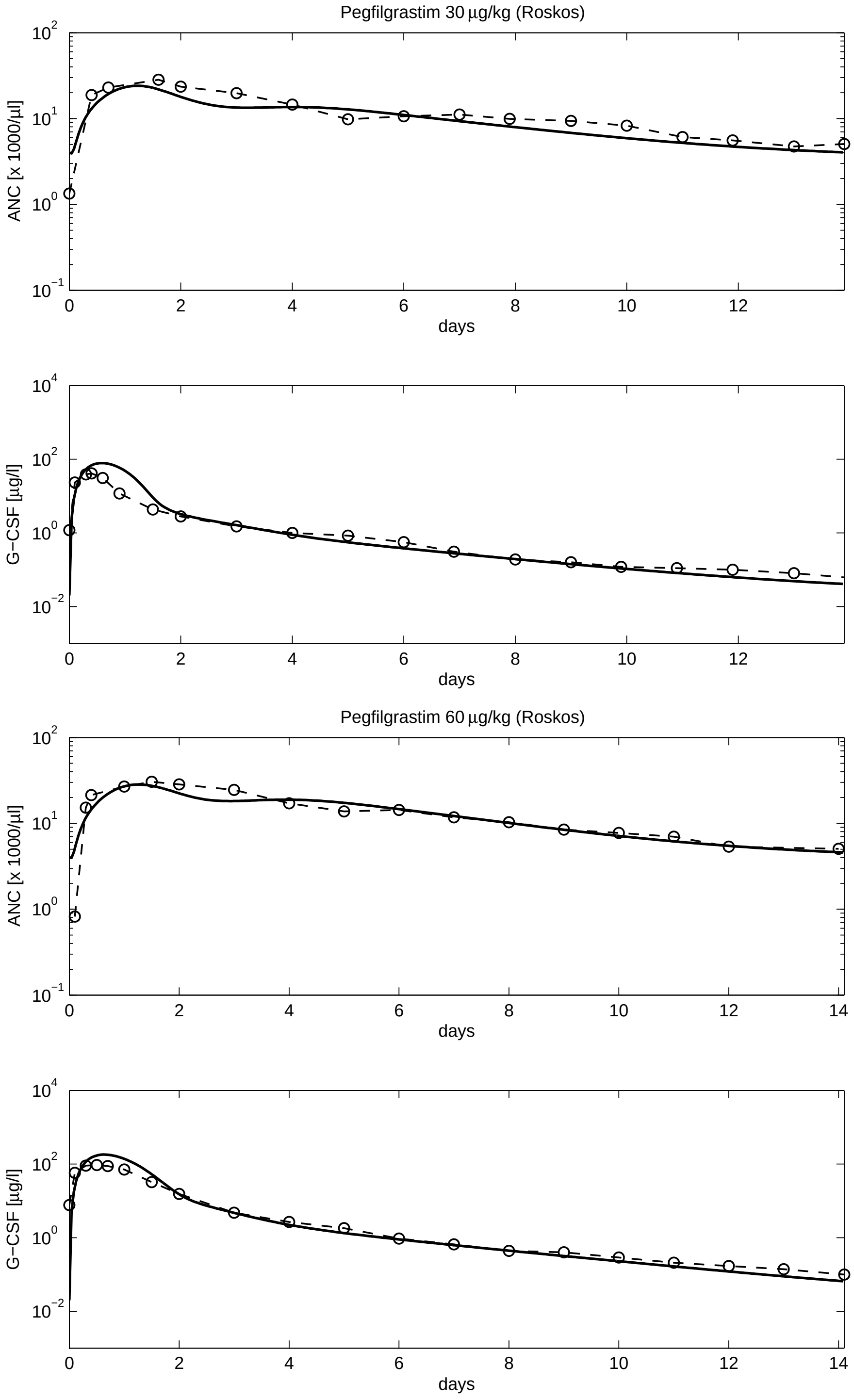
Pegfilgrastim 30 µg/kg (Roskos)
ANC [x 1000/µl]
days
G−CSF [µg/l]
days
Pegfilgrastim 60 µg/kg (Roskos)
ANC [x 1000/µl]
days
G−CSF [µg/l]
days

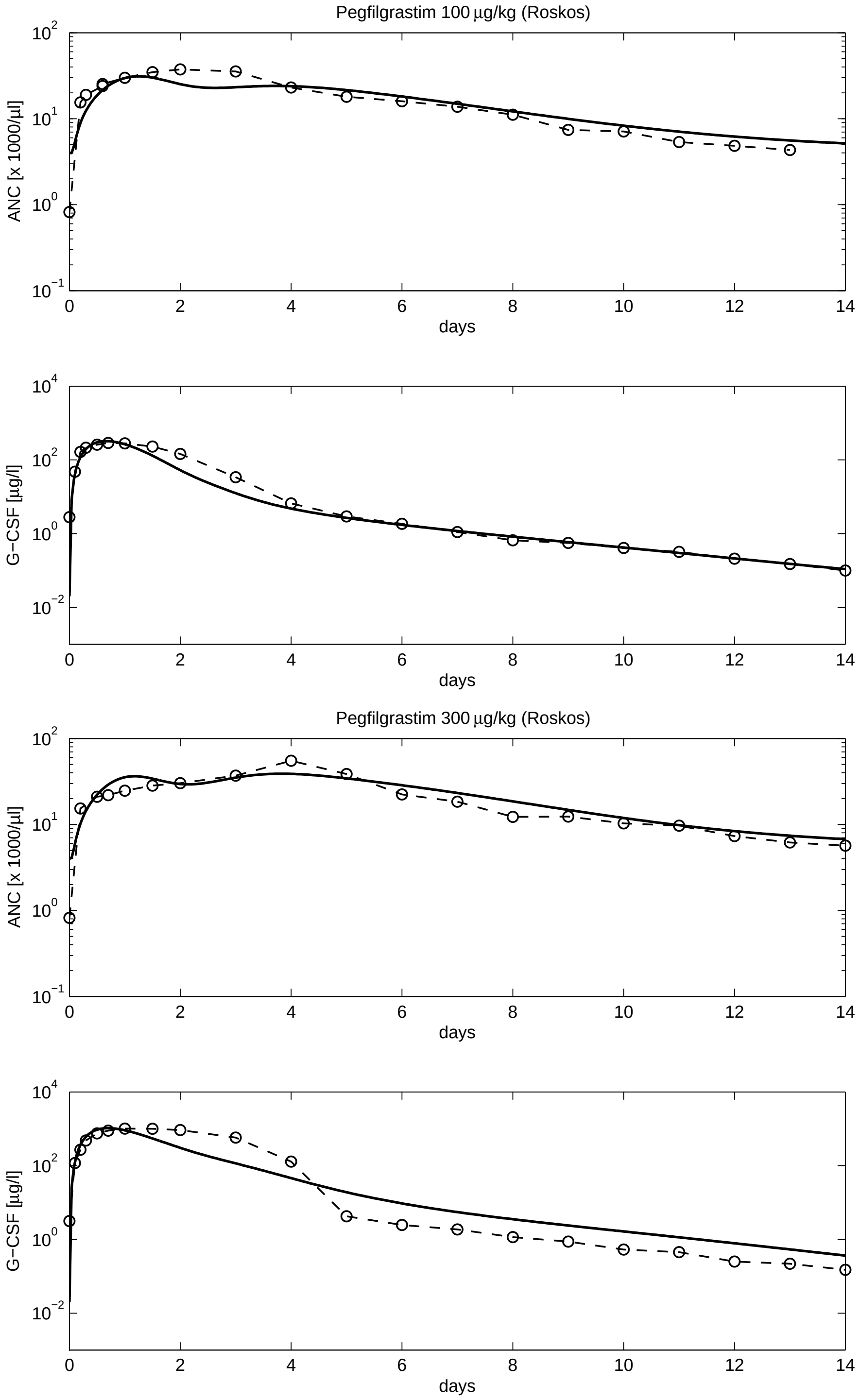

Figure A1: Data sets used for model calibration. Phase I and phase II data sets of G-CSF applications were taken from the literature (dotted lines and circles) and

were compared with the model predictions (solid lines). References are given in table 1. For Johnston *et al.* [6] we only used the data of the first cycle of injections of Filgrastim and Pegfilgrastim which were applied prior to chemotherapy. For injections under chemotherapy, additional toxicity parameters were fitted.

A.4. Sensitivity Analysis

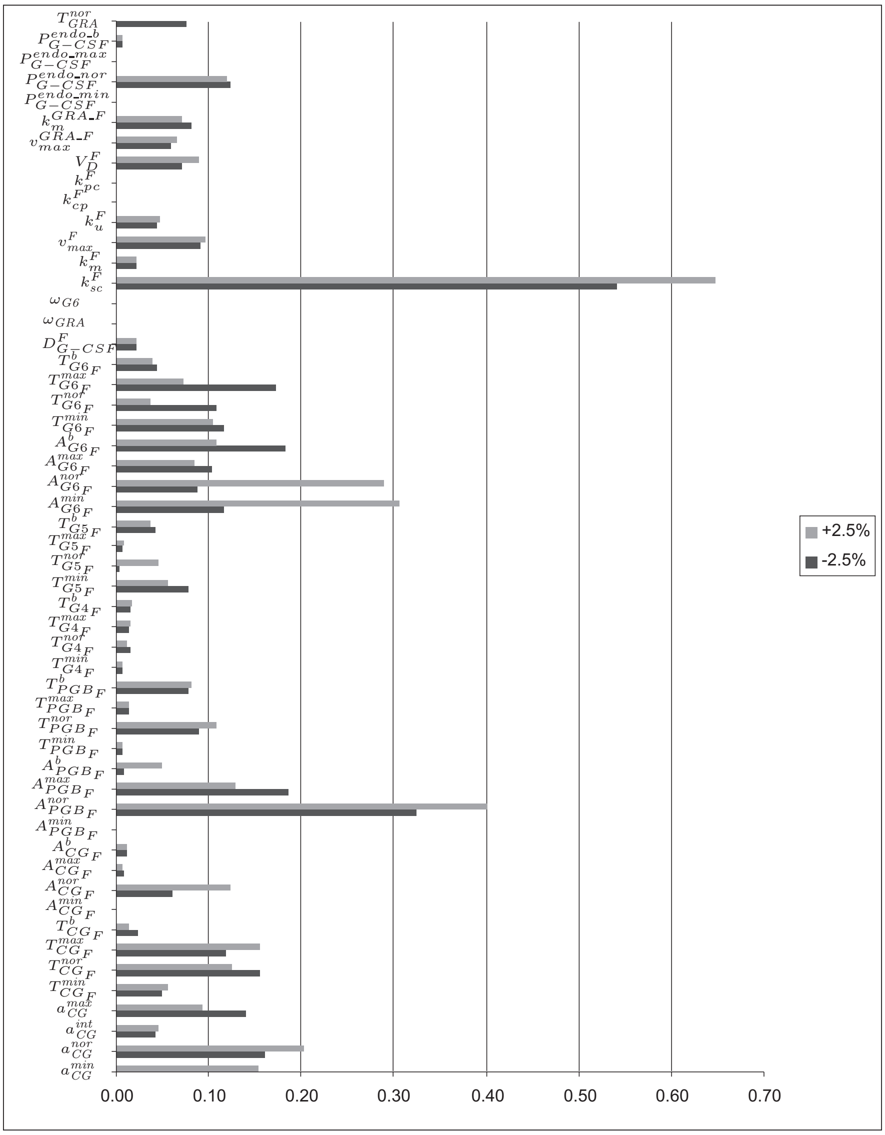

Figure A2: Sensitivity analysis of cell-kinetic parameters and pharmacokinetic and -dynamic parameters of Filgrastim and endogenous G-CSF respectively. Parameters were modified by ±2.5%. Relative change of the fitness function is shown as length of corresponding bars. Meanings of single parameters are explained in section A2.

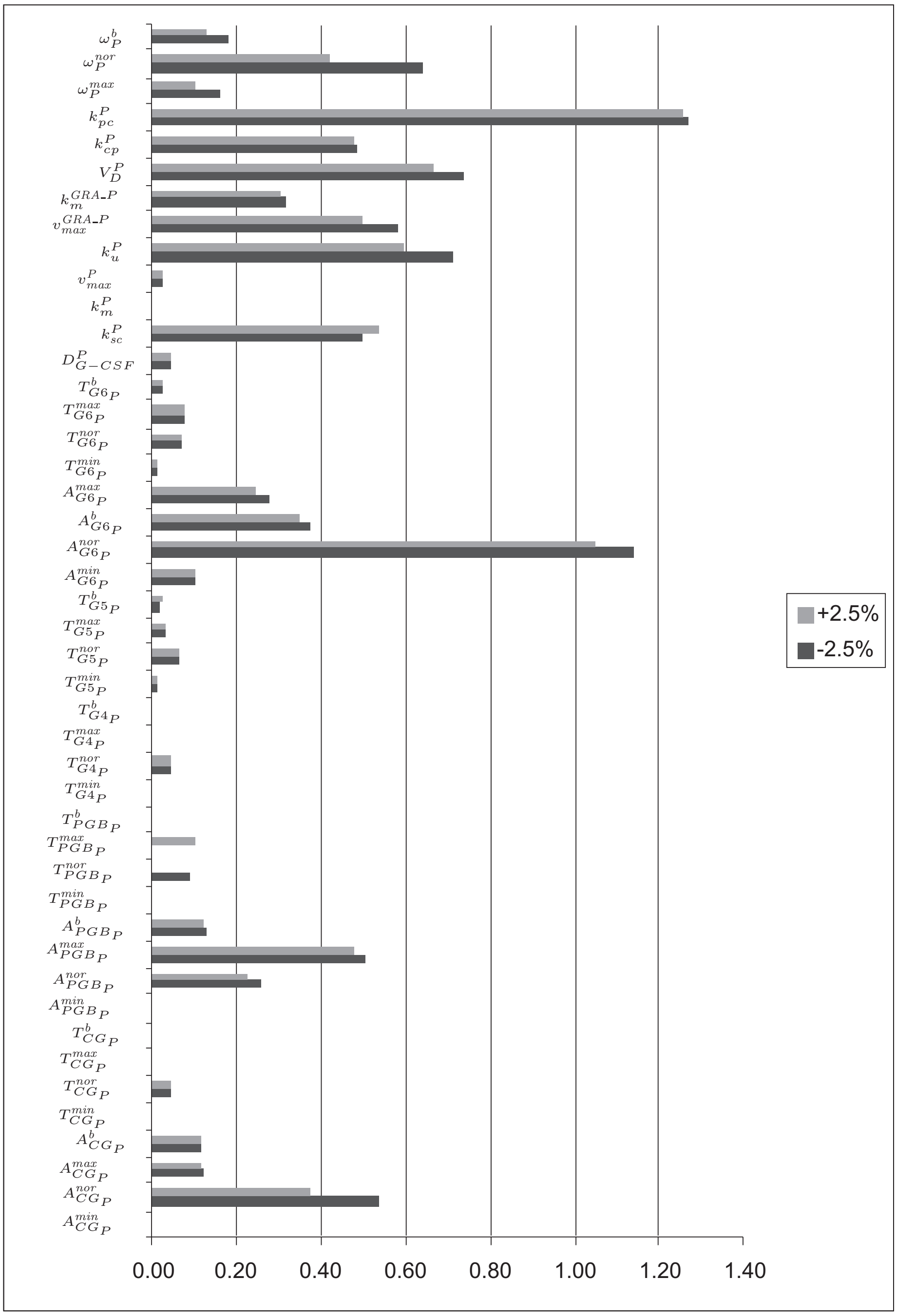

Figure A3: Sensitivity analysis of pharmacokinetic and -dynamic parameters of Pegfilgrastim. Parameters were modified by $\pm 2.5\%$. Relative change of the fitness function is shown as length of corresponding bars. Meanings of single parameters are explained in section A2.

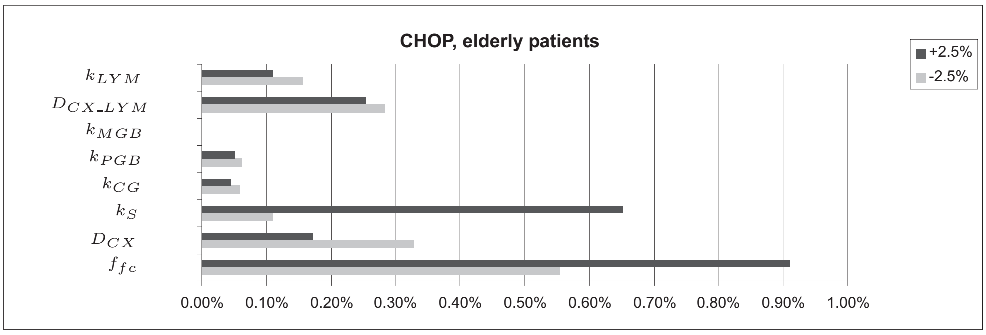

Figure A4: Sensitivity analysis of CHOP chemotherapy parameters. Parameters were modified by ±2.5%. Relative change of the fitness function is shown as length of corresponding bars. Meanings of single parameters are explained in section A2.

References

[1] Scholz, M., Engel, C., Loeffler, M. (2005) Modelling human granulopoiesis under poly-chemotherapy with G-CSF support. *J. Math. Biol.* **50**, 397-439.

[2] Wichmann, H.-E., Loeffler, M. *Mathematical Modeling of Cell Proliferation: Stem Cell Regulation in Hemopoiesis*, CRC Press, Boca Raton 1985.

[3] Mackey, M.C., Aprikyan, A.A., Dale, D.C. (2003) The rate of apoptosis in post mitotic neutrophil precursors of normal and neutropenic humans. *Cell Prolif.* **36**, 27-34.

[4] Schmitz, S., Franke, H., Loeffler, M., Wichmann, H.E., Diehl, V. (1996) Model analysis of the contrasting effects of GM-CSF and G-CSF treatment on peripheral blood neutrophils observed in three patients with childhood-onset cyclic neutropenia Brit. J. Haematol. **95**, 616–625

[5] Dale, D.C., Fauci, A.S., Wolff, S.M. (1974) Alternate-day prednisone. *T.n.E.J.Med.*, 1154–1158.

[6] Johnston, E., Crawford, J., Blackwell, S., Bjurstrom, T., Lockbaum, P., Roskos, L., Yang, B.B., Gardner, S., Miller-Messana, M.A., Shoemaker, D., Garst, J., Schwab, G. (2000) Randomized, dose-escalation study of sd/01 compared with daily filgrastim in patients receiving chemotherapy. *J. Clin. Oncol.* **18**(13), 2522-2528.
